# Advances in the study of extracellular vesicles of *Naegleria fowleri* and their role in contact-independent pathogenic mechanisms

**DOI:** 10.1186/s13071-025-06786-z

**Published:** 2025-05-01

**Authors:** Ismael Castelan-Ramírez, Catalina Flores-Maldonado, Dolores Hernández-Martínez, Lizbeth Salazar-Villatoro, Alberto Daniel Saucedo-Campos, David Segura-Cobos, Adolfo René Méndez-Cruz, Maritza Omaña-Molina

**Affiliations:** 1 Posgrado en Ciencias Biológicas, Universidad Nacional Autónoma de México (UNAM), Coyoacán, Ciudad de Mexico, México; 2 Laboratorio de Amibas Anfizóicas, Carrera de Médico Cirujano, Facultad de Estudios Superiores Iztacala, UNAM, Tlalnepantla, Estado de México México; 3 Departamento de Fisiología, Biofísica y Neurociencias, CINVESTAV, Ciudad de Mexico, México; 4 Departamento de Infectómica y Patogénesis Molecular, CINVESTAV, Ciudad de Mexico, México; 5 Facultad de Estudios Superiores Iztacala, Carrera de Médico Cirujano, UNAM, Tlalnepantla, Estado de México México; 6 Laboratorio de Inmunología, Facultad de Estudios Superiores Iztacala, UNAM, Tlalnepantla, Estado de México México

**Keywords:** Extracellular vesicles, Necrosis, Haemolysis, Proteases, Pathogenicity, *Naegleria fowleri*, Virulence factors

## Abstract

**Background:**

Extracellular vesicles (EVs) are spherical membrane particles released by prokaryotic and eukaryotic cells. EVs produced by pathogenic organisms are known to play a role in host-pathogen interactions; however, despite some reports on *Naegleria fowleri* EVs, their potential role in inducing cytopathic effects remains poorly understood. In this study, we evaluated the role of *N. fowleri* EVs in contact-independent pathogenic mechanisms.

**Methods:**

Extracellular vesicles were characterized via transmission electron microscopy, nanoparticle tracking analysis, SDS-PAGE, mass spectrometry, Western blotting, and zymography. EVs internalization by trophozoites and MDCK epithelial cells was also determined. Finally, mammalian cells were coincubated with EVs to evaluate haemolytic activity, epithelial paracellular ionic permeability alterations, and necrosis.

**Results:**

*Naegleria fowleri* extracellular vesicles, ranging from 82.5 to 576.5 nm in size, were isolated, with a mean of 216.8 nm and a mode of 165.3 nm. Proteomic analysis identified 1006 proteins in the EVs, including leishmanolysin, a protein associated with pathogenic mechanisms such as adhesion and enzymatic processes. The proteolytic activity of EVs was found to be primarily due to serine protease. Furthermore, EVs were internalized by both trophozoites and MDCK cells. Additionally, EVs exhibited haemolytic activity in erythrocytes as well as increased ionic permeability and necrosis in MDCK cells 24 h postinteraction.

**Conclusions:**

*Naegleria fowleri* EVs exhibit proteolytic and haemolytic activity and are internalized by trophozoites and MDCK epithelial cell monolayers, increasing the ionic permeability of the monolayer and inducing necrosis. Furthermore, these vesicles contain molecules associated with pathogenic processes such as leishmanolysin. Our results suggest that EVs facilitate paracellular invasion, migration, and damage caused by trophozoites and play a significant role in pathogenic processes as part of a contact-independent mechanism, which, in conjunction with a contact-dependent mechanism, enhances our understanding of the pathogenicity exhibited by this amphizoic amoeba during its invasion of target tissues.

**Graphical abstract:**

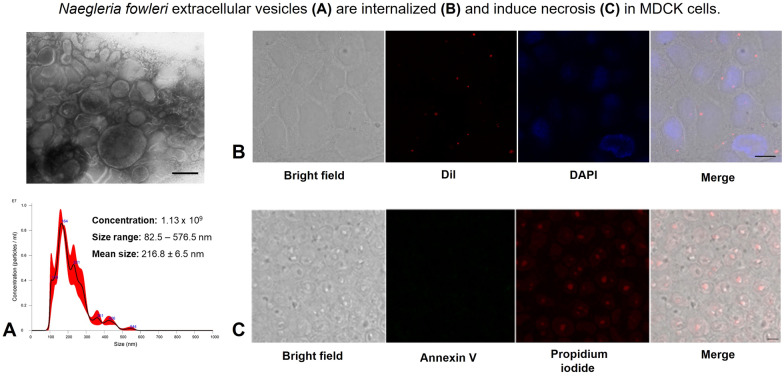

**Supplementary Information:**

The online version contains supplementary material available at 10.1186/s13071-025-06786-z.

## Background

*Naegleria fowleri* is a thermophilic and ubiquitous protozoan found in air, soil, and warm waters that plays a significant role in the biological control of bacterial populations and contributes to the enrichment of soil with nitrogen and phosphorus, which are essential nutrients for plants. The life cycle of this protozoan consists of three stages: the trophozoite stage, which is the feeding, motile, and reproductive stage; the temporary flagellar stage, which occurs in response to sudden environmental changes; and the resistant cystic stage [1–3]. *Naegleria fowleri* is also known as an amphizoic amoeba because of its ability to exist freely in nature and as a parasite. In humans, it is the causative agent of primary amebic meningoencephalitis (PAM), an acute and fulminant haemorrhagic infection that is usually reported in children and young adults who were recently exposed to contaminated water. PAM is characterized by a mortality rate of approximately 95%, which is attributed to the acute nature of the infection, delays in diagnosis, and lack of effective treatments [2–5]. The *Naegleria fowleri* trophozoite enters through nasal passages, adheres to the nasal epithelium, crosses the cribriform plate via the olfactory nerves, and spreads to the olfactory bulbs [3–6].

To date, the pathogenic mechanisms carried out by these protozoa during their invasion of target tissues, including contact-dependent and -independent mechanisms, have been partially described. In vitro studies have shown that amoebae adhere to cells via glycoproteins with mannose residues and then migrate toward tight junctions, altering ZO-1 and claudin-1 proteins. Finally, amoebae invade other tissue areas where they carry out phagocytic processes through amoebostomes, events known as contact-dependent mechanisms [5–8]. Reports on the contact-independent pathogenic mechanisms of *N. fowleri* include the secretion of proteases with mucinolytic activity [9], cysteine proteases that disrupt tight junctions [10], pore-forming proteins [11, 12], and the release of electron-dense granules [13].

All types of cells, including protozoa, release EVs, which are defined as spherical particles surrounded by a lipid bilayer containing complex molecules that actively participate in intercellular communication; regarding pathogens, EVs facilitate the transfer of virulence factors [14, 15]. EVs are classified into three main subtypes depending on their size and biogenesis: exosomes (30–150 nm) generated from multivesicular bodies; microvesicles or ectosomes (100–1000 nm), formed by budding or evagination of the plasma membrane; and apoptotic bodies, which form during cell apoptosis [16].

Analysing the role that EVs play in other extracellular parasitic protozoa, it has been reported that EVs released by the highly adherent *Trichomonas vaginalis* strain can increase the adhesion of strains with low adhesive capacity [17]. In addition, de Souza Gonçalves et al. [18] reported that *Acanthamoeba castellanii* EVs induce necrosis in the Chinese hamster ovary (CHO) cell line, whereas *A. culbertsoni* EVs induce haemolysis [19]. *Naegleria fowleri* secretes EVs, which exhibit immunomodulatory effects on various cells [20–24]; however, whether they induce any cellular damage has yet to be demonstrated.

In this study, *N. fowleri* (ATCC 30808) EVs were characterized by their morphology, proteomic and proteolytic profiles, intraspecies interactions, and interspecies interactions with both MDCK cells and trophozoites after their internalization, which may play a role in transferring virulence factors and facilitating communication. Moreover, the EVs induced a cytopathic effect on erythrocytes and MDCK epithelial cells, suggesting an important role in contact-independent pathogenic mechanisms.

## Methods

### *Naegleria fowleri* culture

The reference strain of *N. fowleri* (ATCC 30808), which has been maintained with high virulence through serial mouse passages, was used in this study. Cultures were grown in 75-cm^2^ culture flasks containing 2% bactocasitone medium supplemented with 10% fetal bovine serum (FBS) and 1% antibiotics (penicillin-streptomycin) at 37 °C. All assays were performed using trophozoites in the exponential growth phase.

### Obtaining extracellular vesicles

EVs were obtained from 90% confluent amoebic cultures containing approximately 14 × 10^6^ trophozoites per 75-cm^2^ flask. Briefly, bactocasitone medium supplemented with FBS was removed from the cultures, and the flasks were washed with sterile PBS (pH 7.4). Then, 8 ml of bactocasitone medium, free of FBS and antibiotics, was added, and the cultures were incubated at 37 °C for 24 h. This incubation period ensured optimal conditions for the trophozoites and maximized EVs harvest, a method previously used with other *Naegleria* strains to obtain vesicles [20]. Depleting FBS prevents vesicle contamination [16]. Subsequently, EVs were recovered according to Sierra-López et al. [19]: after 24 h, the culture supernatant was sequentially centrifuged at 400×*g* for 10 min to remove trophozoites and then at 1300×*g* for 10 min to eliminate cellular debris. The supernatant was filtered through a 1.2-µm pore membrane and centrifuged at 16,800×*g* for 40 min at 4 °C. The resulting EVs pellet was washed twice with PBS.

### Characterization of extracellular vesicles

#### Transmission electron microscopy (TEM)

EVs were observed by negative staining, following the standard method previously described [19]. Secreted EVs (5 μl) were pipetted onto the surface of Formvar-coated copper grids (400 mesh). The samples were dried with filter paper and stained with 2.5% uranyl acetate for 20 s. Grids were air-dried and carbon-coated in a vacuum evaporator (JEE400, JEOL Ltd., Tokyo, Japan). The samples were examined using a JEM-1011 transmission electron microscope, a Gatan Orius SC1000A1 camera, and Gatan Digital Micrograph software version 2.30.542.

#### Nanoparticle tracking analysis (NTA)

For NTA, EVs were diluted 1:2000 in 0.22-µm membrane-filtered PBS. A NanoSight NS300 equipped with an sCMOS camera and NTA 3.2 software (Dev Build 3.2.16) were used to determine the EVs concentration and size distribution. The detection threshold was set to 8, the camera level was set to 13, and the defocus distance and maximum hopping distance were set automatically. Triplicate measurements were performed at 25 °C.

#### Observation of EVs released by *N. fowleri* trophozoites

To confirm the release of EVs by trophozoites and characterize their morphology via TEM, the samples were processed as previously described [19]. Briefly, 1 × 10^7^ trophozoites were placed in a Petri dish in 10 ml bactocasitone medium without FBS and incubated for 2 h at 37 °C to promote amoeba adhesion. Afterward, the medium was recovered, and trophozoites and EVs were harvested by centrifugation at 16,800 × g for 40 min. The pellets were then fixed with 2.5% glutaraldehyde in 0.1 M sodium cacodylate buffer (1 h), pH 7.2, followed by postfixation with 1% osmium tetroxide in 0.1 M sodium cacodylate buffer. The samples were dehydrated with increasing concentrations of ethanol, transferred to propylene oxide, and embedded in epoxy resin, which was polymerized at 60 °C for 24 h. Ultrathin sections (60 nm) were obtained, subsequently contrasted with uranyl acetate and lead citrate, and finally observed under a Jeol JEM-1011 transmission electron microscope (JEOL Ltd., Tokyo, Japan).

#### Determination of the protein profile of *N. fowleri* trophozoites and EVs via SDS-PAGE

Trophozoites and EVs were collected separately in PBS supplemented with a protease inhibitor cocktail (Roche, Cat. 11836153001) and then lysed by freeze-thaw cycles (5 for trophozoites, 3 for EVs). The samples were mixed with loading buffer containing β-mercaptoethanol (1:4) and boiled for 4 min. Twenty micrograms of EVs or trophozoite proteins were separated on a 12% polyacrylamide gel (100 V, 120 min).

#### Immunorecognition of *N. fowleri* EVs via Western blotting

Antigen detection in *N. fowleri* EVs via Western blotting was performed. Proteins separated by 12% SDS-PAGE were electrotransferred to PVDF membranes under semidry conditions (18 V, 60 min). The membrane was blocked with 5% fat-free milk in TBST for 1 h and incubated overnight at 4 °C with rabbit anti-*N. fowleri* polyclonal serum (1:2500 dilution). After being washed with TBST, the membrane was incubated with alkaline phosphatase-conjugated goat anti-rabbit IgG (1:5000) (Invitrogen, Ref. 31340, USA) for 1.5 h at room temperature. The membrane was washed three times with TBST, and antigenic proteins were detected via Novex® AP chromogenic substrate (BCIP/NCP) (Invitrogen, Ref. WP20001, Life Technologies Corp., Carlsbad, CA, USA).

#### Liquid chromatography-mass spectrometry (LC-MS) analysis of *N. fowleri* EVs

EVs equivalent to 130 μg of protein were subjected to SDS-PAGE on a 12% gel and separated and concentrated into a small zone ~ 1 cm into the resolving gel; then, the gel was stained with Coomassie G-250 (Bio-Rad). The resulting band was cut under sterile conditions and subjected to “in-gel” trypsin digestion [25, 26]. The generated tryptic peptides were separated on an HSS T3 C18 column (Waters, Milford, MA); 75 μm × 150 mm, 100 A° pore size, 1.8 μm particle size; and a UPLC ACQUITY M-Class (Waters, Milford, MA). Mobile phase A consisted of 0.1% formic acid (FA) in water, and mobile phase B consisted of 0.1% FA in acetonitrile with the following gradient: 0 min, 7% B; 121.49 min, 40% B; 123.15 to 126.46 min, 85% B; 129 to 130 min, 7% B; and a flow rate of 400 nl min^−1^ at 45 °C on the column [27]. The spectral data were acquired with a Synapt G2-Si mass spectrometer with electrospray ionization and ion mobility separation (Waters, Milford, MA) via a data-independent acquisition (DIA) approach in high-definition multiplexed MS/MS mode (HDMSE). The ionization was set with the following parameters: 2.75 kV in the sampler capillary, 30 V in the sampling cone, 30 V in the source offset, 70 °C for the source temperature, 0.5 bar for the nanoflow gas, and 150 l/h for the purge gas flow. The precursor ions were fragmented in the transfer cell via a collision energy ramp from 19 to 55 eV.

The raw files containing MS and MS/MS spectra were analysed by ProteinLynx Global Server (PLGS) v3.0.3 software [28] (Waters) via a target decoy strategy [29] against *N. fowleri* *. fasta database (obtained from UniProt, UP000444721, 13764 protein sequences). The parameters used for protein identification were as follows: trypsin as the cutting enzyme and one missed cleavage allowed; carbamidomethyl (C) as a fixed modification and oxidation (M), amidation (C-terminal), deamidation (Q, N), or phosphorylation (S, T, Y) as variable modifications; peptide and fragment tolerance were set to automatic, minimum fragment ion matches per peptide: 2, minimum fragment ion matches per protein: 5, minimum peptide matches per protein: 1, and false discovery rate at 1%. All identifications had a percentage of ≥ 95% reliability (Protein AutoCurate green). Gene Ontology analysis was performed via the PANTHER GO platform. The mass spectrometry proteomics data were deposited at the ProteomeXchange Consortium via the PRIDE [1] partner repository with the dataset identifier PXD059563.

### Biological activity of EVs

#### Internalization of EVs by *N. fowleri* trophozoites

To determine whether EVs are internalized by *N. fowleri* trophozoites, TEM and confocal microscopy were performed.

The TEM assay procedure was performed in Petri dishes as previously described. To perform the confocal microscopy experiments, purified EVs were stained red with the lipophilic dye Dil (3 µM) (Invitrogen, Ref: D3911, USA) for 10 min in the dark at room temperature, followed by two washes with PBS. In parallel, 2 × 10^6^ *N. fowleri* trophozoites were placed in 500 µl of FBS-free bactocasitone medium on coverslips in 24-well plates and incubated for 30 min with the stained EVs (released by approximately 14 × 10^6^ amoebae). The samples were subsequently fixed with 4% paraformaldehyde for 20 min and washed three times with PBS, and the nuclei were stained with DAPI (300 nM) (SIGMA, Ref. D9542, USA) for 10 min. Finally, the samples were mounted and observed through a confocal laser scanning microscope (Leica TCS SP8, Leica Camera, Wetzlar, Germany) and processed with Leica Microsystems CMS GmbH version 3.5.21594.6.

#### Internalization of EVs from *N. fowleri* by Madin-Darby Canine Kidney (MDCK) epithelial cells

To evaluate EVs internalization, MDCK epithelial cells (clone 7.15) were seeded to confluence on a coverslip in DMEM supplemented with 10% FBS and antibiotics. The cultures were incubated at 37 °C in a 5% CO_2_ atmosphere. After 24 h, the cells were washed three times and incubated for 2 h with serum-free DMEM before being exposed to Dil-stained vesicles for 30 min at 37 °C. The samples were then fixed with 4% paraformaldehyde and washed three times with PBS, and the nuclei were stained with DAPI (300 nM) (SIGMA, Ref. D9542, USA) for 10 min. Finally, the samples were mounted and observed under a confocal laser scanning microscope (Leica TCS SP8, Leica Camera, Wetzlar, Germany), with images processed with Leica Microsystems CMS GmbH version 3.5.21594.6.

#### Evaluation of the proteolytic activity of *N. fowleri* EVs

Zymograms were generated to evaluate whether the EVs exhibited proteolytic activity. Briefly, 10 µg of protein from EVs and total extracts from trophozoites were separated via 8% SDS-PAGE and copolymerized with 0.4% porcine skin gelatin. Electrophoresis was conducted at 4 °C and 100 V for 2 h.

After electrophoresis, the gels were washed with a 1% Triton X-100 solution for 30 min with orbital agitation to remove the SDS. After being incubated overnight (12 h) in buffer containing 50 mM Tris-HCl and 10 mM CaCl_2_, pH 7.0, at 37 °C, the gels were washed with distilled water and stained with Coomassie Brilliant Blue R-250. Unstained areas of the gel represent proteolytic activity.

The EVs were incubated for 45 min at 37 °C with specific inhibitors prior to electrophoresis to identify the type of proteases in the vesicles: 2 mM phenylmethylsulfonyl fluoride (PMSF) for serine proteases, 2 mM iodoacetamide (IA) for cysteine proteases, or 10 mM EDTA for metalloproteases.

The proteolytic activity of the different samples was compared via semiquantitative densitometric analysis (ImageJ software). The assays were performed in triplicate.

#### Haemolytic activity of *N. fowleri* EVs

To analyse whether *N. fowleri* EVs could lyse erythrocytes, haemolytic activity assays were performed according to those carried out by Pierson et al. [30] and Sierra-Lopez et al. [19], who evaluated the haemolytic activity of *Francisella novicida* and *A. culbertsoni* EVs, respectively. The implemented method has the advantages of being inexpensive, accessible, and simple to perform. If EVs cause haemolysis, haemoglobin (along with other erythrocyte constituents) is released into the supernatant, increasing the absorbance of samples, which was determined using a standard spectrophotometer [31]. Briefly, 40 µg of protein contained in EVs was added to 1 ml of PBS containing 2% human blood erythrocytes (Type O Rh +) in suspension, with or without a serine protease inhibitor (2 mM). Erythrocytes incubated in PBS were considered the negative control, and erythrocytes in distilled water were considered the positive control. All samples were incubated at 37 °C for 4 h. Then, all samples were centrifuged at 1000×*g* for 2 min to eliminate cells, and the absorbance of the supernatants was measured at 540 nm. The experiments were performed in triplicate. The formula used to calculate the haemolysis percentage is shown below:

$$\left( {\frac{{{\text{Absorbance of sample}} - {\text{Absorbance of no haemolysis}}}}{{{\text{Absorbance of positive control }} - {\text{Absorbance of total haemolysis}}}}} \right) \times 100$$

Absorbance of the negative control = Absorbance of no haemolysis.

Absorbance of positive control = Absorbance of total haemolysis.

#### The effect of *N. fowleri* EVs on epithelial paracellular ionic permeability in MDCK cells

To determine whether EVs affect the paracellular ionic permeability of the MDCK cell line, transepithelial electrical resistance (TER) was measured in monolayers exposed to EVs. MDCK cells are known to replicate key epithelial features, including the formation of a continuous monolayer [32], tight junction development [33, 34], junctional complexes, plasma membrane polarity, and transepithelial transport [35, 36]. Furthermore, the development and maintenance of tight junctions can be experimentally evaluated by estimating TER when cultured on semipermeable supports [34, 36].

Briefly, 8.25 × 10^4^ MDCK cells were cultured on Transwell™ semipermeable inserts in DMEM supplemented with 10% FBS and antibiotics. The cultures were incubated at 37 °C in a 5% CO_2_ atmosphere. After 48 h, the inserts were transferred to serum-free DMEM for 24 h, followed by incubation with 40 μg of *N. fowleri* EVs proteins. Simultaneously, assays were performed with a serine protease inhibitor (2 mM PMSF) to assess the potential participation of these enzymes in TER modification. The TER of the monolayers was recorded with the EVOM2™ system. The experiments were performed in triplicate.

#### Analysis of induced cell death in the MDCK epithelial cell line by *N. fowleri* EVs

To determine whether EVs induce cell death, interactions with the MDCK cell line were performed. Briefly, 2.5 × 10^5^ MDCK cells/cm^2^ were cultured on coverslips in DMEM supplemented with 10% FBS and antibiotics and incubated at 37 °C in a 5% CO_2_ atmosphere. After 48 h, the coverslips were incubated for 24 h in serum-free DMEM. Subsequently, 40 μg of vesicle protein, with or without serine protease inhibitor (2 mM PMSF), was washed with PBS and added to the MDCK monolayers. As positive controls for apoptosis and necrosis, staurosporine (1 μg/ml) [37] and 20 mM hydrogen peroxide were used, respectively.

At 24 h postinteraction, the monolayers were fixed with 4% paraformaldehyde and processed to detect apoptosis or necrosis using an Annexin V-FITC/Propidium Iodide Detection Kit (Sigma-Aldrich, St. Louis, MO) following the manufacturer's instructions. Finally, the samples were observed under a confocal laser scanning microscope (Leica TCS SP8, Leica Camera, Wetzlar, Germany), and images were processed with Leica Microsystems CMS GmbH version 3.5.21594.6. Necrotic cells were also quantified in at least three fields per condition (0.75×).

### Statistical analysis

Statistical analysis was performed via GraphPad Prism 5.0 software. One-way analysis of variance (ANOVA) followed by Tukey's multiple comparison test was used to analyse the data. Values of *P* < 0.05 were considered statistically significant.

## Results

### EVs characterization

**Fig. 1:**
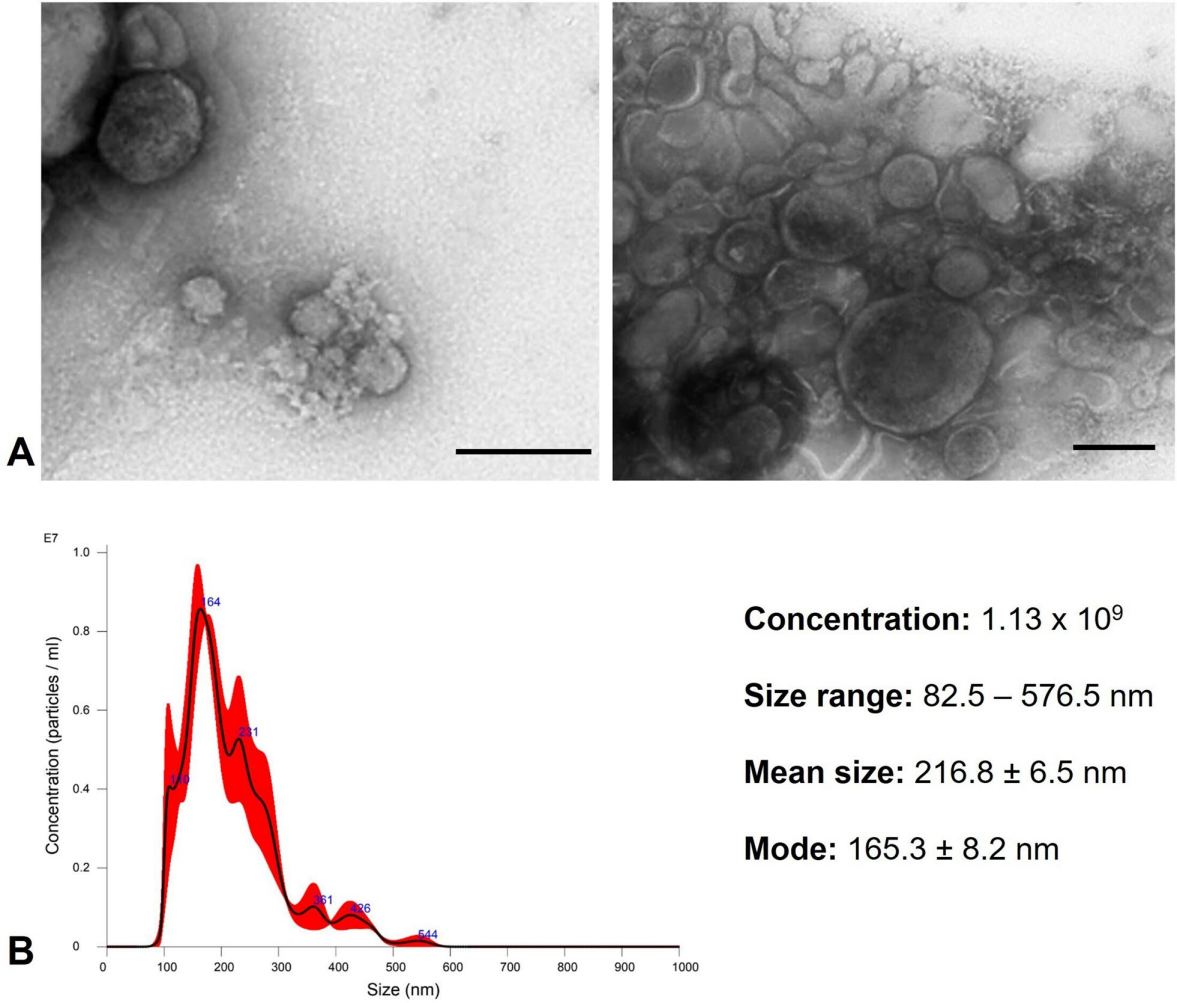
Analysis of extracellular vesicles of *Naegleria fowleri.* **A** Through TEM, EVs, which are delimited by a membrane, were observed. Scale bar = 250 nm. **B** EV size and concentration were determined using NanoSight NS300 equipment. A graphical representation of the size and concentration of extracellular vesicles released by trophozoites after 24 h of incubation is shown

EVs from *Naegleria fowleri* (ATCC 30808) were isolated following the MISEV 2018 and MISEV 2023 guidelines [14, 16]. *Naegleria fowleri* EVs were obtained 24 h postincubation of trophozoites in bactocasitone medium without FBS. TEM with negative staining revealed that the EVs exhibited a characteristic morphology: spherical particles delimited by a membrane (Fig. 1A). NTA revealed that a total of 1.13 × 10^9^ vesicles were released by 3 × 10^7^ *N. fowleri* trophozoites, with a size distribution ranging from 82.5 nm to 576.5 nm. The average vesicle size was 216.8 ± 6.5 nm, and the mode was 165.3 ± 8.2 nm (Fig. 1B). Both TEM and NTA provided consistent results regarding the size of the EVs, with a broad size distribution suggesting the presence of a heterogeneous population of EVs.

#### Observation of EVs emission by *N. fowleri* trophozoites

**Fig. 2:**
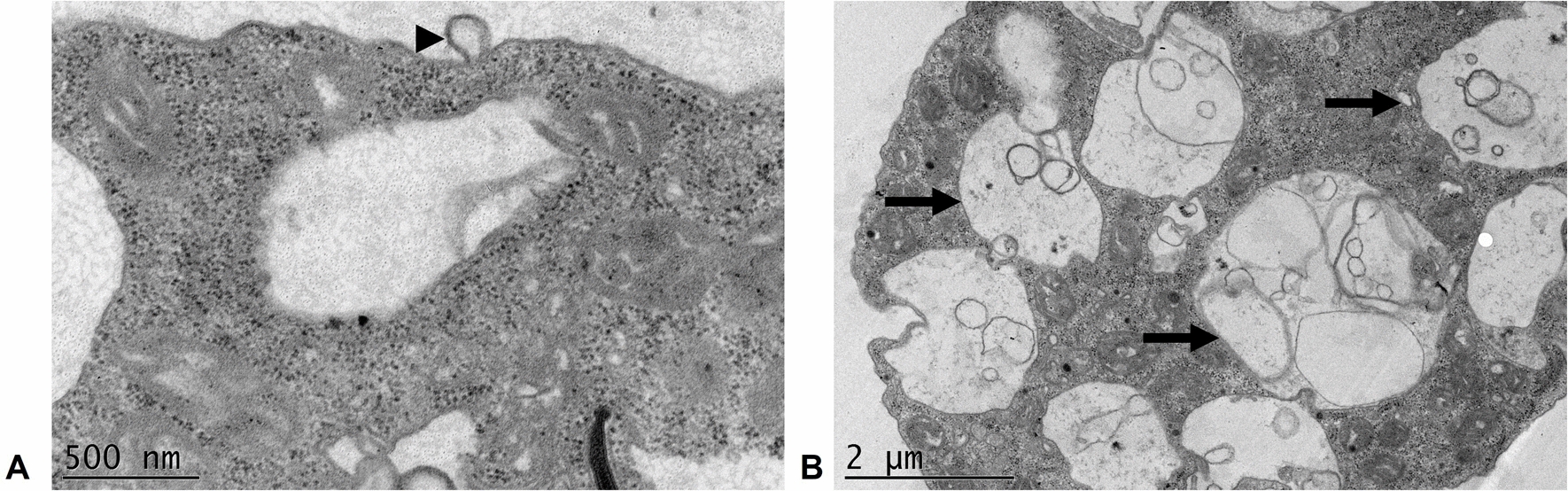
Probable release of extracellular vesicles by *N. fowleri* trophozoites. TEM. **A** Membrane evagination (arrowhead) suggests the formation of microvesicles. **B** Several structures, indicated by arrows, appear to be multivesicular bodies, which are cellular precursors involved in exosome secretion

Electron micrograph analysis revealed plasma membrane evaginations in *N. fowleri* trophozoites (Fig. 2A), suggesting the release of microvesicles. Additionally, the presence of multivesicular bodies within the cytoplasm of the trophozoites were observed (Fig. 2B), which strongly suggests exosome production, as these structures are key intermediates in the biogenesis of these particles.

#### Protein profiling via electrophoresis and the evaluation of protein recognition via Western blotting

**Fig. 3:**
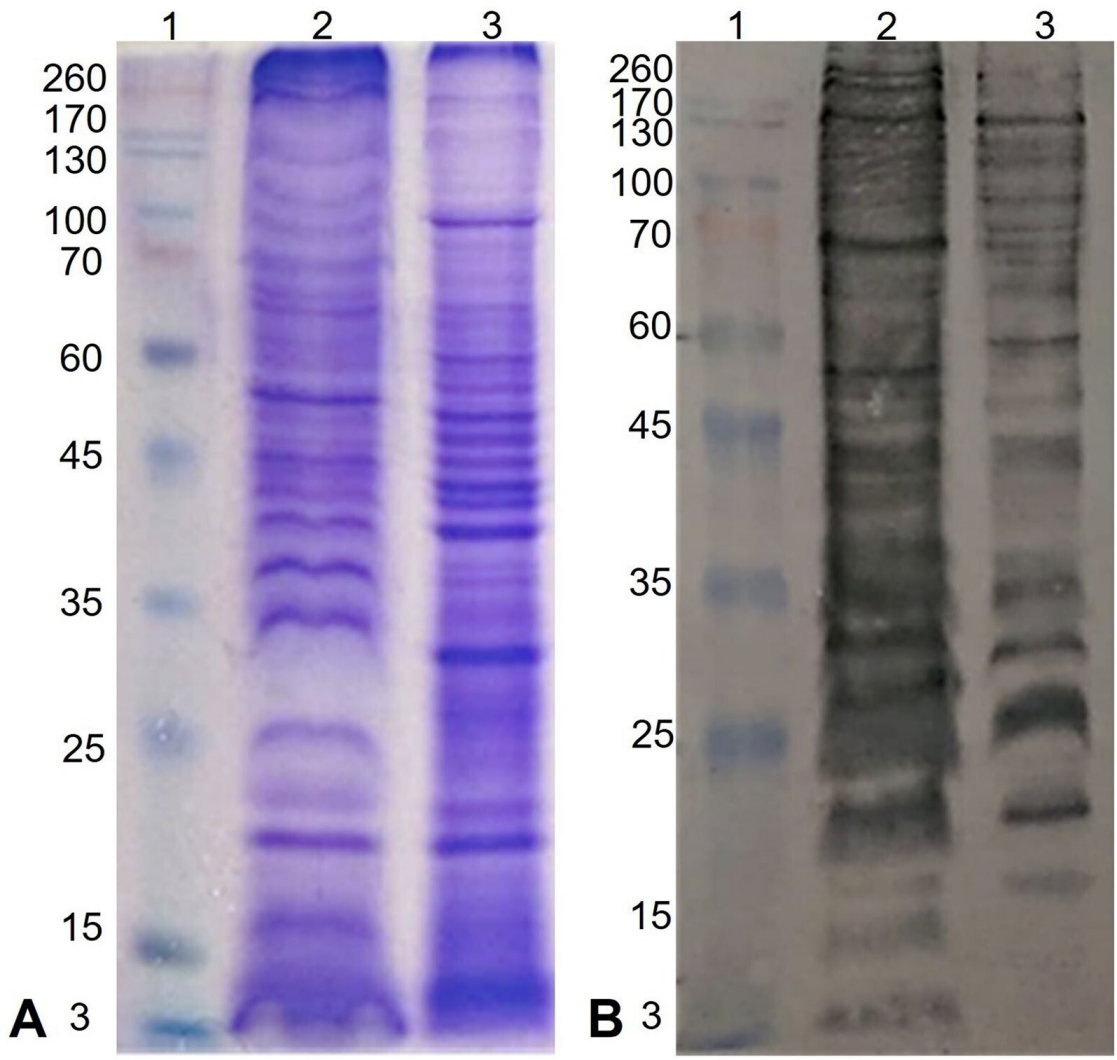
Protein profiling and protein recognition of *N. fowleri* extracellular vesicles. **A** Coomassie blue staining. Protein bands ranging from 3 to 260 kDa were observed; however, notable differences between the protein recognition patterns of trophozoites and extracellular vesicles were observed. **B** Western blot analysis. Different proteins were recognized between extracellular vesicles and trophozoites. Lane 1: Molecular weight marker. Lane 2: *Naegleria fowleri* extracellular vesicles. Lane 3: *Naegleria fowleri* trophozoite extract

The protein profiles of *N. fowleri* trophozoites and EVs are shown in Fig. 3A. Both trophozoites and EVs contain a broad range of proteins, with molecular weights ranging from 3 to 260 kDa, with some overlapping protein bands. However, notable differences in protein profiles between the two groups were observed. For example, a 32-kDa protein present in trophozoites was absent in the vesicles, whereas proteins with molecular weights of approximately 25 kDa, 35 kDa, and 38 kDa were significantly enriched in the EVs.

Western blot analysis using polyclonal anti-*N. fowleri* antibodies confirmed the presence of *N. fowleri* proteins in both trophozoites and EVs, further corroborating the observed differences. The Western blot results revealed a broader range of protein recognition in EVs, ranging from 3 to 260 kDa, whereas protein recognition in trophozoites was predominantly confined to the range of approximately 20 kDa to 200 kDa (Fig. 3B).

#### LC-MS analysis of *N. fowleri* EVs

**Fig. 4:**
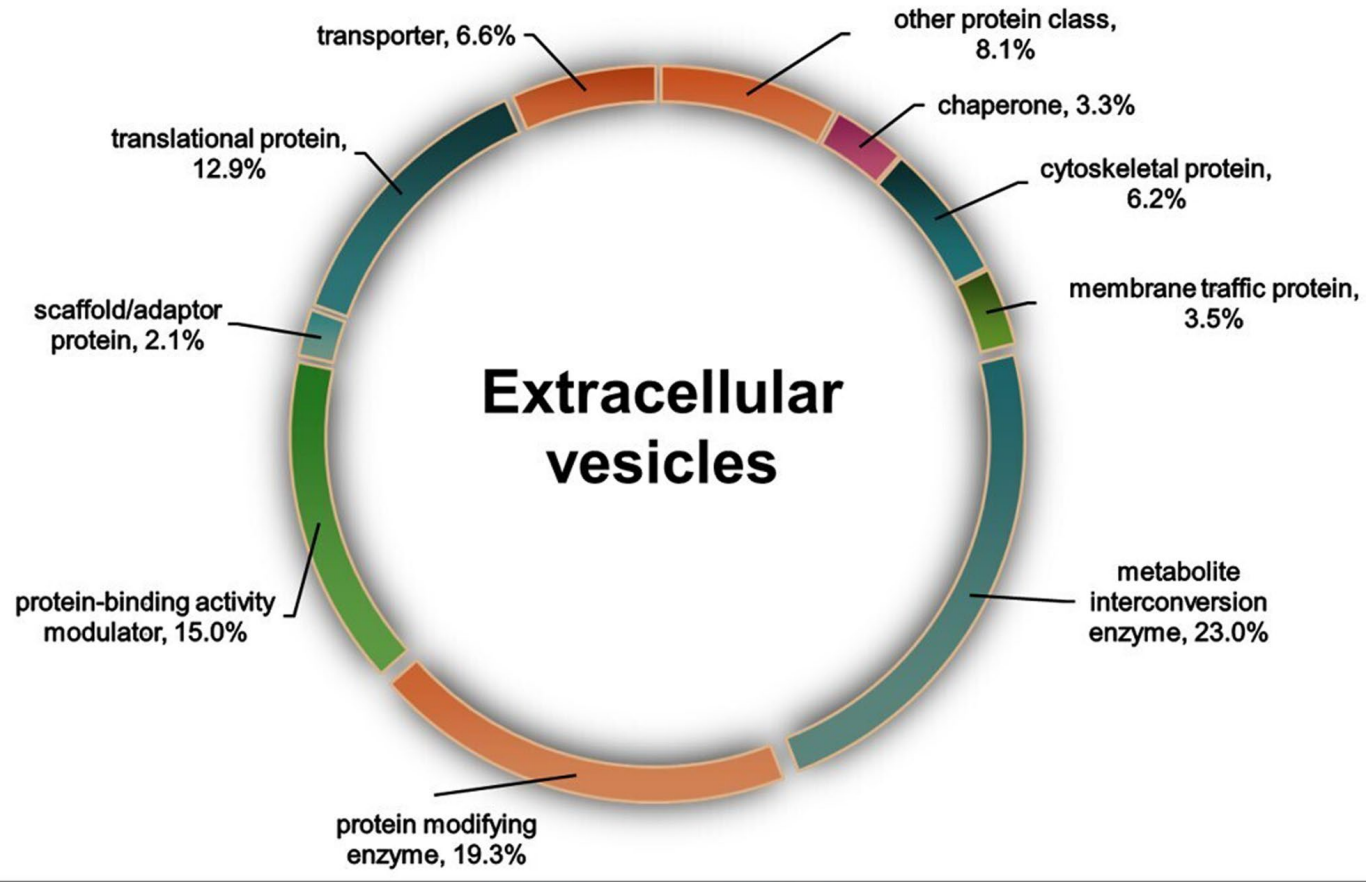
Main protein groups contained in the extracellular vesicles of *Naegleria fowleri*. Proteomic analysis of EVs, performed via the PANTHER GO platform, identified metabolite interconversion enzymes and protein-modifying enzymes as the most abundant protein groups

EVs secreted by *N. fowleri* trophozoites were analysed via mass spectrometry. A total of 1006 proteins were identified, of which 202 are still uncharacterized in the current UniProt database. The identified proteins were analysed on the PANTHER GO platform, which revealed that, within the protein class scheme, those with the highest prevalence were metabolite interconversion enzymes (23%), protein-modifying enzymes (19.3%), protein-binding activity modulators (15%), translational proteins (12.9%), transporters (6.6%), cytoskeletal proteins (6.2%), membrane trafficking proteins (3.5%), chaperones (3.3%), scaffold/adaptor proteins (2.1%), and other protein classes with low prevalence but also important (8.1%) (e.g. RNA metabolism proteins, extracellular matrix proteins, calcium-binding proteins, defense/immunity proteins, transfer/carrier proteins, chromatin/chromatin-binding proteins, regulatory proteins, DNA metabolism proteins, gene-specific transcriptional regulators, cell adhesion proteins, intercellular signal molecules, and transmembrane signal receptors) (Fig. 4, Additional file 1: Fig. S1).

In addition, the molecular functions of 675 proteins were identified, proteins with catalytic activity (41.8%), being the most prevalent, followed by binding proteins (34.5%) (Additional file 2: Fig. S2A). Regarding biological processes, 882 proteins were identified, 37.9% of which were associated with cellular processes and 30.2% with metabolic processes (Additional file 2: Fig. S2B). Finally, 688 proteins related to cellular components were identified and classified into two categories: cellular anatomical entities (72.7%) and protein-containing complexes (27.3%) (Additional file 2: Fig. S2C).

Importantly, when consulting the list of proteins commonly reported in exosomes (http://exocarta.org/exosome_markers_new), we found that *N. fowleri* vesicles contain 6 proteins of the 32 most frequently reported proteins (Additional file 3: Table S1). Notably, 70-kDa heat shock protein (HSP 70) and glyceraldehyde-3-phosphate dehydrogenase (GAPDH) are among the most frequently reported proteins in exosomes. Finally, proteomic analysis revealed the presence of some biologically significant proteins, including leishmanolysin and members of the calpain family.

### Biological activity of EVs

#### Internalization of EVs by *N. fowleri* trophozoites

**Fig. 5:**
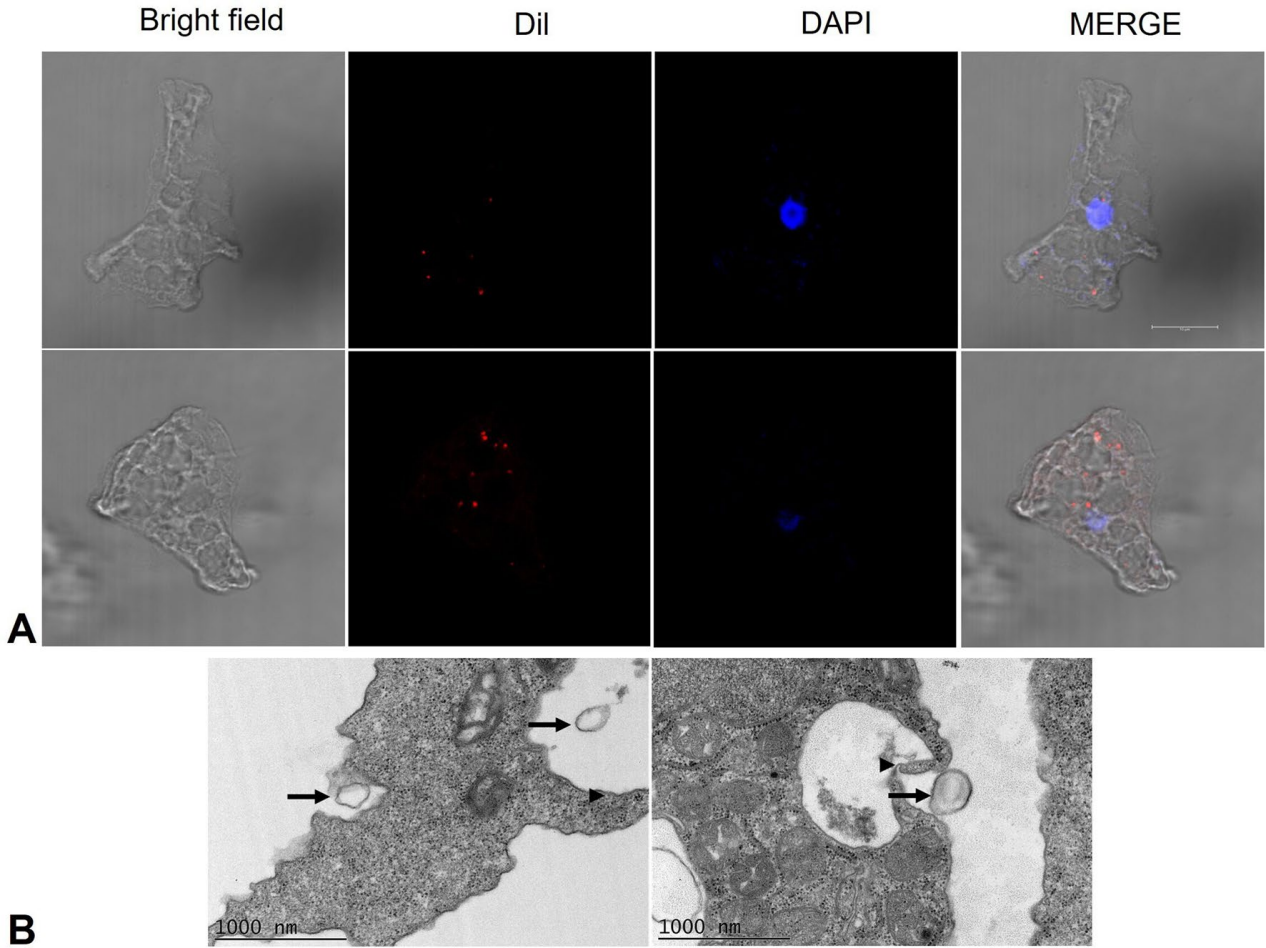
Internalization of extracellular vesicles by *Naegleria fowleri* trophozoites. **A** Confocal microscopy revealed Dil-stained EVs in red within the cytoplasm of trophozoites, indicating internalization. Scale bar = 10 µm. **B** Transmission electron microscopy. Cytoplasmic extensions of *N. fowleri* trophozoites (arrowheads) are observed surrounding particles that are likely extracellular vesicles (arrows)

To investigate whether *N. fowleri* trophozoites internalize EVs, both confocal microscopy and transmission electron microscopy were performed. Confocal microscopy images revealed the presence of Dil-stained EVs within the cytoplasm of the amoebae, which appeared as distinct red puncta (Fig. 5A). This observation suggests the successful internalization of EVs by trophozoites. The results were further supported by TEM, which provided higher resolution images showing the close proximity of EVs to the trophozoite surface. Notably, cytoplasmic extensions were observed reaching the EVs (Fig. 5B).

#### Internalization of EVs by MDCK cells

**Fig. 6:**
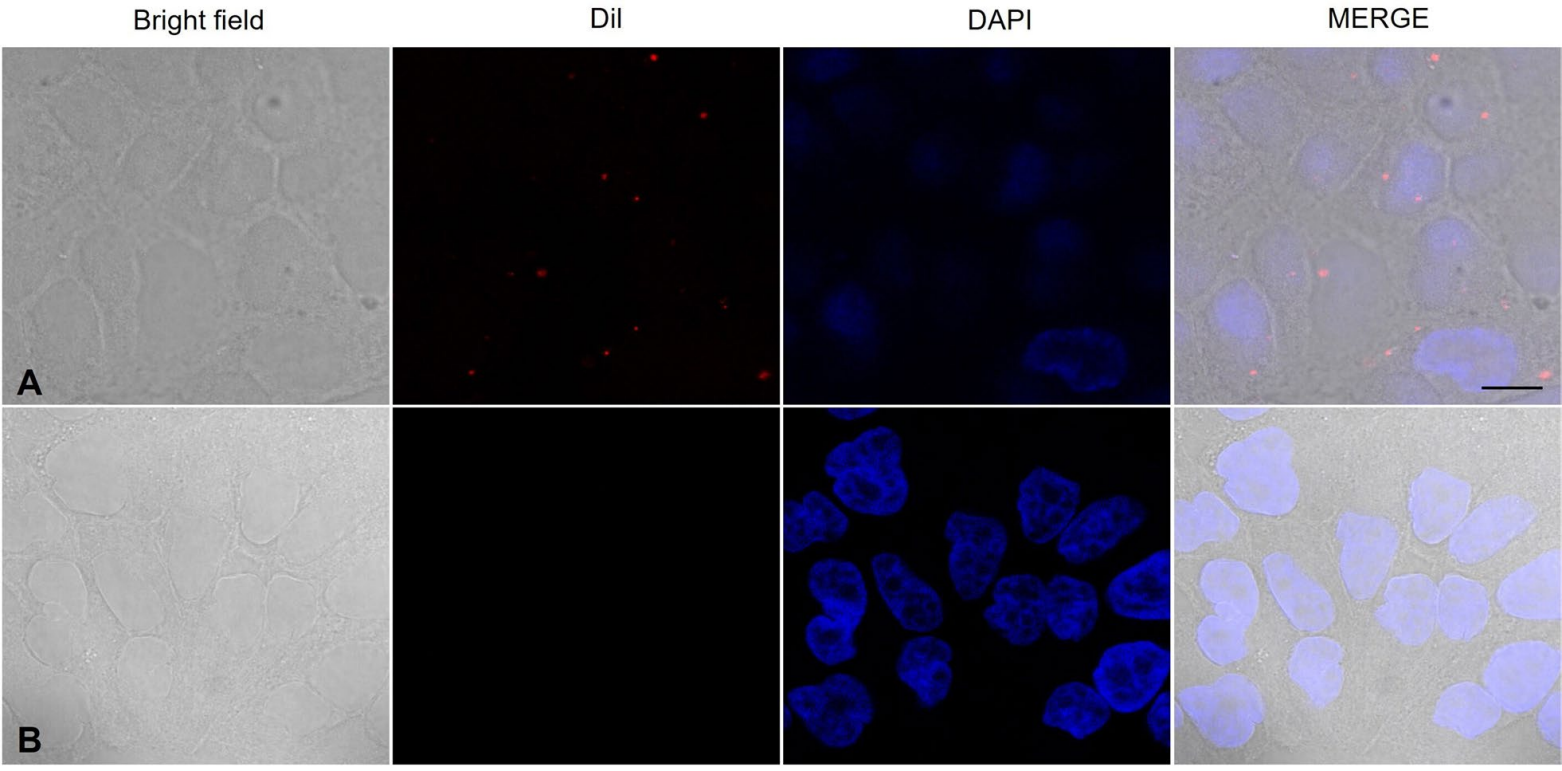
Internalization of extracellular vesicles by MDCK cells. Confocal microscopy. **A** Dil-stained EVs are observed within the cytoplasm of MDCK cells, indicating successful internalization. **B** No intracellular fluorescence signal was detected in the control culture. Scale bar = 10 µm

To assess the potential internalization of EVs by mammalian epithelial cells, experiments were conducted using the MDCK cell line and Dil-labeled EVs, which were visualized via confocal microscopy. After 30 min of interaction, EVs were observed within the cytoplasm of the MDCK cells (Fig. 6A). A control experiment was performed simultaneously, where the MDCK cells were processed under the same conditions but without the addition of EVs. The control images, shown in Fig. 6B, demonstrated the absence of puncta and red fluorescent signals within the cells, confirming that the fluorescence observed in Fig. 6A was due to the internalization of the labeled vesicles.

#### Proteolytic activity

**Fig. 7:**
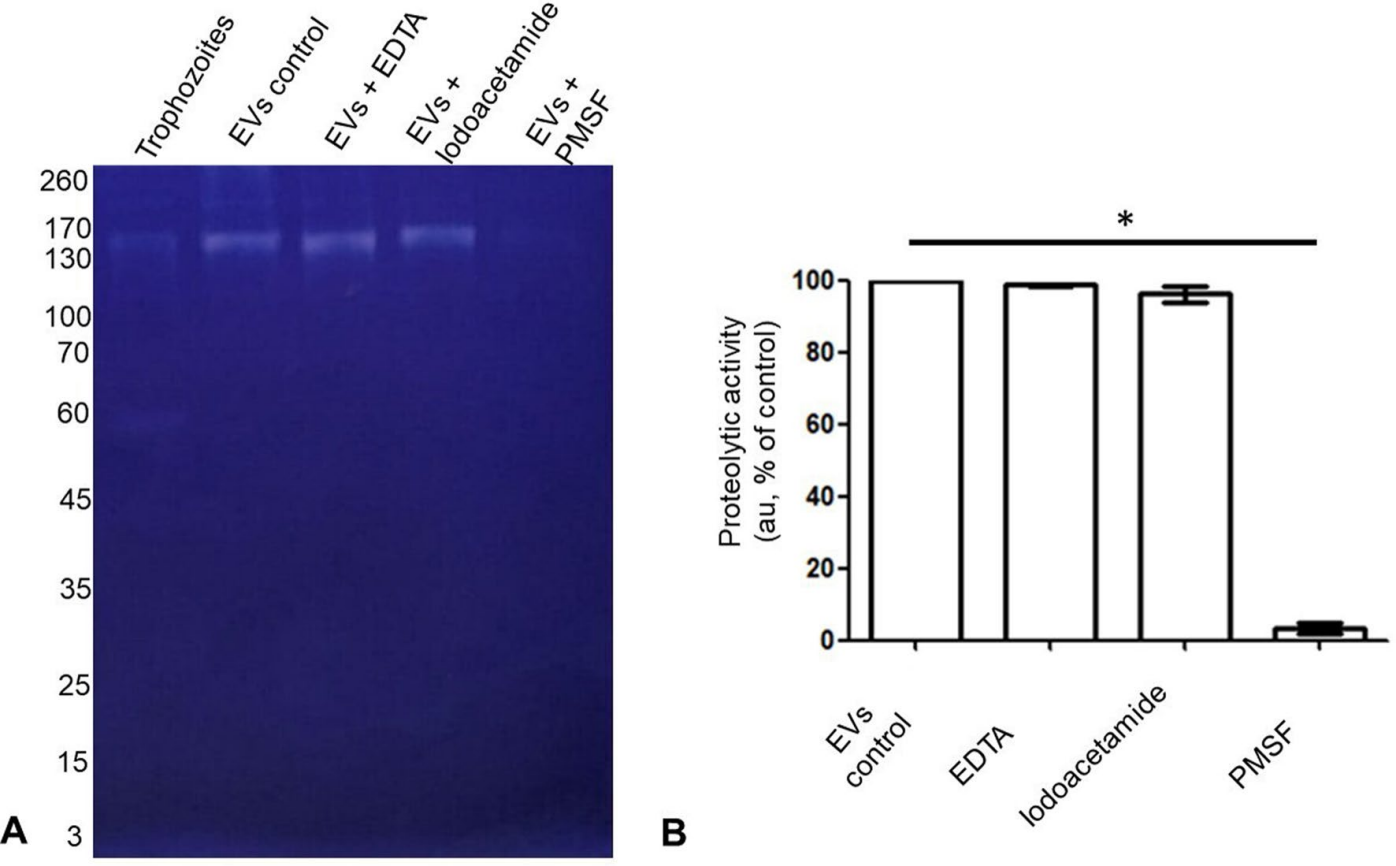
Proteolytic activity of EVs and total trophozoite extract. **A** Unstained areas of the zymogram, ranging from approximately 60 to 260 kDa for trophozoites and 100 to 260 kDa for EVs, were observed, indicating gelatin degradation by proteolytic activity. **B** Zymogram densitometric analysis. A greater reduction in proteolytic activity was observed with PMSF

Zymograms were generated to characterize the proteolytic activity of *N. fowleri* EVs. The vesicles contain proteolytic enzymes, with molecular weights ranging from approximately 100 to 260 kDa. However, the total trophozoite extract contained enzymes with molecular weights ranging from 60 to 260 kDa. A significant depletion of proteolytic activity was observed when the samples were incubated with the serine protease inhibitor (Fig. 7A, line 5). This decrease was confirmed by densitometric analysis (Fig. 7B).

#### Haemolytic activity

Haemolysis was assessed by incubating red blood cells with EVs and analysing the amount of haemoglobin released using a spectrophotometer [38]. The absorbance values revealed a significant difference in the percentage of haemolytic cells induced by *N. fowleri* EVs (mean: 24.8% ± SD 3.6) compared with that of the negative control, which presented a much lower value (mean: 4% ± SD 1.9). However, when EVs were incubated with a serine protease inhibitor, the percentage of haemolytic cells remained similar (mean: 22.2% ± SD 5.6), indicating that the presence of the protease inhibitor did not significantly alter the haemolytic effect of the EVs (Additional file 4: Fig. S3). This increase in haemolysis by EVs suggests an important cytotoxic property.

#### Effect of *N. fowleri* EVs on the paracellular ionic permeability of MDCK cells

The transepithelial electrical resistance of monolayers exposed or not exposed to EVs was measured. After 24 h of interaction, the TER decreased (mean: 234.3 Ω ± SD 10) compared with that of the control (mean: 316.7 Ω ± SD 14.4), suggesting that EVs increase ionic flow through tight junctions. Notably, a similar result was observed when the EVs were incubated with a serine protease inhibitor (mean: 230 Ω ± SD 8.6) (Additional file 5: Fig. S4).

#### EVs induce cell death in epithelial MDCK cells

**Fig. 8:**
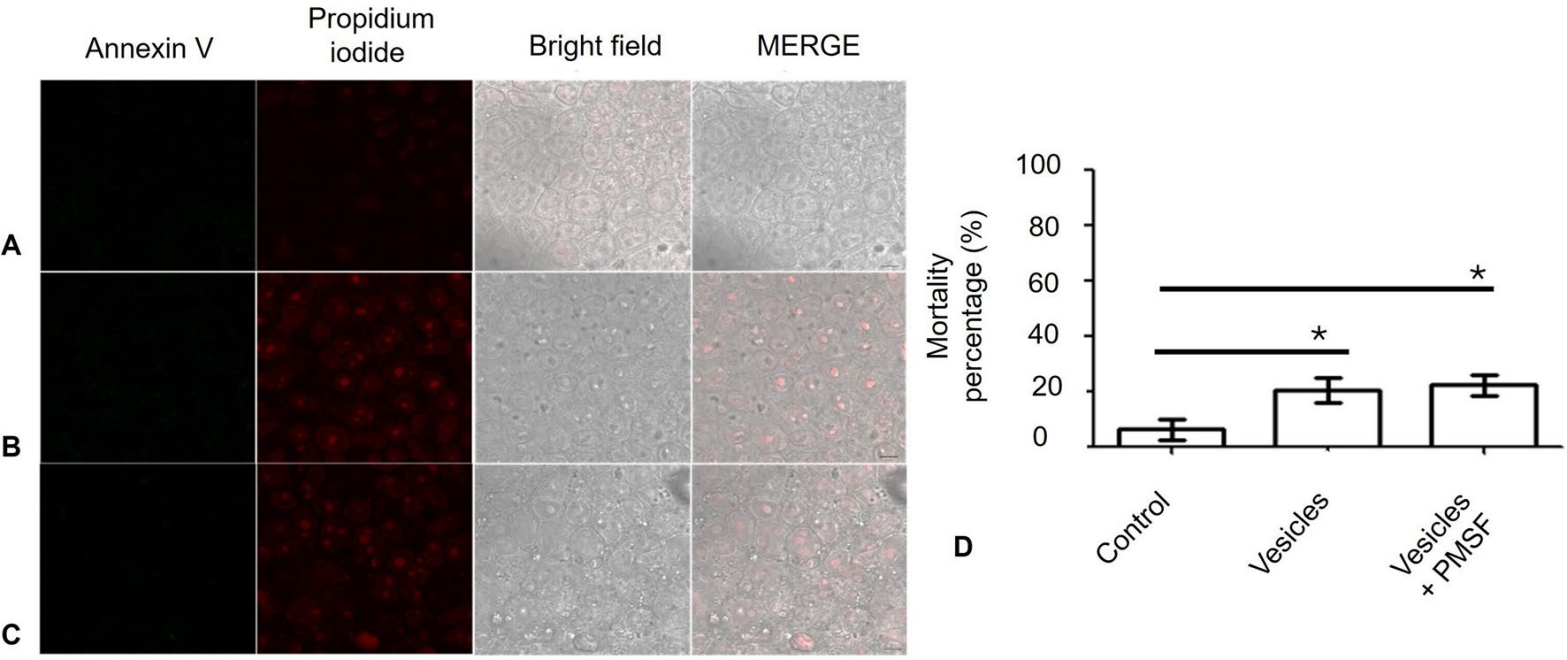
*Naegleria fowleri* EVs induce necrosis but not apoptosis in MDCK cells. Apoptosis and necrosis were assessed through Annexin V (green) and propidium iodide (red) staining, respectively. **A** Control culture: no evidence of apoptosis or necrosis was observed. **B** MDCK cells underwent necrosis following interaction with EVs. **C** EVs induced necrosis in MDCK cells, even when they were incubated with PMSF. **D** Percentage of necrotic cells in cultures (*P < 0.05); *n* = 3. Bar = 20 µm

To assess whether the vesicles induced cell death in MDCK cells, interactions were conducted under controlled experimental conditions. At 24 h postinteraction, no evidence of cell death was observed in the control group (Fig. 8A), indicating that the cells remained viable under the experimental conditions. However, when the MDCK cells interacted with EVs, there was clear evidence of necrosis (Fig. 8B), indicating that EVs may contribute to cellular damage. Additionally, EVs induced necrosis even when a protease inhibitor is present (Fig. 8C).

Necrotic cells were quantified from at least three visual fields of cells in each group. Significant differences were observed between the control cells and the MDCK cells exposed to EVs, regardless of the presence of the protease inhibitor (Fig. 8D).

## Discussion

*Naegleria fowleri* is the causative agent of PAM, an acute infection that primarily affects children and young adults, with a mortality rate exceeding 95%. Understanding the pathogenic mechanisms of this protozoan is essential for developing pharmacological strategies to treat this pathology. While several mechanisms involved in *N. fowleri* pathogenicity, such as adhesion, migration, phagocytosis, and extracellular vesicle secretion, have been partially characterized, the role of *N. fowleri* EVs in inducing cellular damage remains unexplored [6, 20, 24]. This study describes the morphological, proteomic, and proteolytic features of *N. fowleri* (ATCC 30808) EVs. Furthermore, EVs were internalized by both trophozoites and MDCK cells, leading to an increase in ionic permeability and necrosis 24 h postinteraction. Additionally, EVs also showed haemolytic activity in erythrocytes.

Vesicles were isolated from 24 h cultures, a time point that ensured both amoebic integrity and optimal EVs production [20]. These particles predominantly exhibited a spherical morphology with a lipid bilayer and diameters ranging from 82.5 to 576.5 nm, a size range consistent with those reported for other *N. fowleri* strains [20, 21]. In addition, according to the literature [39], two likely EVs release pathways were observed via TEM (Fig. 2): direct budding from the trophozoite membrane and exosome release via multivesicular bodies.

Regarding the EVs content, SDS-PAGE revealed a protein profile that differed from that reported in previous studies, possibly because of variations in factors such as environmental amoebic culture conditions, vesicle isolation methods, and the strain being studied [21]. Both our work and that reported by Retana et al. [21] revealed a 260-kDa protein, which is notably similar in size to the lectin reported in *Entamoeba histolytica* [40]. Further studies are needed to identify the protein corresponding to this molecular weight. Additionally, future experiments should modify electrophoresis conditions to detect a broader range of protein sizes, possibly by using an acrylamide gradient gel.

The protein profile of EVs varies significantly from that of trophozoites, likely because of selective packaging during vesicle biogenesis [41]. In addition, the absence of certain trophozoite proteins in the vesicles could imply that these proteins are not involved in vesicle-mediated functions. The 25-, 35-, and 38-kDa proteins, which are highly concentrated in EVs despite their low expression in trophozoites, may be involved in host-pathogen interactions or cellular communication.

Similarly, the results of our Western blot analysis differed from those reported by Retana et al. [21], since in this study EVs exhibited more bands recognized by anti-*N. fowleri* antibodies. The presence of a wide variety of antigenic molecules in EVs released by *N. fowleri* further reinforces their role as virulence factors, as more than 15 molecules involved in invasion, including mucolytic, adhesin, and lipolytic activities, among others, have been described [6]. EVs with antigenic molecules could be relevant in the pathogenesis of PAM, a disease characterized by an intense host immune response that induces nasal epithelial damage and the penetration and invasion of deeper layers of the epithelium, allowing access to the central nervous system (CNS) [6, 42].

A total of 1006 proteins were identified in the EV analysis in this study, which is greater than the 184 proteins reported for the EVs from a strain of *N. fowleri* isolated from a patient [21] and less than the 2270 proteins reported by Russell et al. [23] for another *N. fowleri* clinical isolate. As previously mentioned, these differences could be explained by variations in EV isolation methods, strain virulence, or time and culture conditions [21, 23]. Despite this, our proteomic results align with those previously reported by Russell et al. [23], who also identified metabolite interconversion enzymes and protein-modifying enzymes as the predominant protein classes in *N. fowleri* EVs.

Among the identified proteins, we also detected leishmanolysin/GP63, a protein in *Leishmania* associated with virulence and pathogenesis [43]. This protein has multiple functions, including metalloprotease, adhesin, and COX-like activities [44]. Leishmanolysin-like proteins have also been identified in parasitic protozoans such as *A. castellanii*, *E. histolytica*, *Giardia duodenalis*, and *Trypanosoma cruzi* [45]. Furthermore, leishmanolysin-like proteins have been reported in *A. culbertsoni* EVs, which exhibit COX activity [19], as well as in *Leishmania* exosomes, which are relevant for the process of cutaneous leishmaniasis infection, suggesting that these proteins play a role in other parasites [46]. Although a leishmanolysin-like protein was recently reported in *N. fowleri* trophozoites [45], this study is the first to highlight EVs leishmanolysin in this amphizoic amoeba. This discovery suggests that the protein may be involved in key pathogenic processes, including adhesion, enzymatic activity, and exacerbation of proinflammatory responses. COX may contribute to the exacerbation of inflammation by generating prostaglandins that amplify the inflammatory response [47]. During *N. fowleri* infection, severe inflammation may be one of the main contributors to irreversible brain damage. These processes potentially contribute to the pathogenesis of PAM.

Nonetheless, although the presence of DNA in small vesicles from *N. fowleri* has been recently reported [24], determining the complete composition of genetic material, lipids, and carbohydrates contained within EVs to elucidate their biological role is essential.

 Zymogram analysis demonstrated that the proteolytic activity of *N. fowleri* EVs was different from that observed in trophozoites, suggesting that protease secretion via EVs plays a key role in *N. fowleri* pathogenicity. Proteases are involved in the mucinolytic degradation of the olfactory epithelium, facilitating adherence, invasion [6, 9], and degradation of the extracellular matrix [6, 48] and tight junction proteins such as ZO-1 and claudin-1 [8], processes in which EVs may be involved. In addition, the results also suggest that the vesicles mostly contain serine proteases, as evidenced by the decreased proteolytic activity upon the addition of the PMSF inhibitor. Our findings are consistent with those of previous reports on *N. fowleri* EVs, where the proteolytic activity of EVs was shown to be due mainly to their serine protease content [21].

Additionally, it has been reported that EVs move through the extracellular medium over time [49], playing an important role in intraspecific communication. For example, EVs released by drug-resistant *Leishmania* strains carry resistance genes that can be transferred to drug-sensitive strains [50]. In contrast, highly adherent *T. vaginalis* secretes EVs that, when interacting with poorly adherent strains, enhance their adhesion properties [17]. In our study, we demonstrated that *N. fowleri* vesicles can be internalized by trophozoites of the same species, suggesting an intraspecific communication process, which could favor, among other factors, the transmission of virulence factors. EV-mediated communication can occur through various mechanisms, such as phagocytosis by recipient cells, vesicle fusion with cell membranes, or activation of cellular receptors [51]. The observation of trophozoite membrane extensions surrounding extracellular vesicles supports the hypothesis that intraspecies communication occurs via EVs phagocytosis.

Through confocal microscopy, we observed the internalization of *N. fowleri* EVs by MDCK cells, a finding consistent with previous reports showing the internalization of EVs from other *N. fowleri* strains by mouse neuroblastoma cells [23], glial cells (C6), and microglia (BV-2) [22]. Specifically, it has been suggested that macrophages phagocytose EVs [20].

The PAMPs (pathogen-associated molecular patterns) expressed by *N. fowleri* are still unknown; however, it is possible that lipopeptidophosphoglycans or lectins, as observed in *E. histolytica* [52–54], may play a role in vesicle internalization. EVs PAMPs can be recognized by nonopsonic cell surface receptors [55]. Further studies are needed to elucidate the mechanism of vesicle internalization.

One of the most important findings of this study is that *N. fowleri* EVs can induce cellular damage, as the interaction of EVs with human erythrocytes leads to their lysis. These results are consistent with findings from Pierson et al. [30], who reported haemolytic activity in *F. novicida* vesicles, and Sierra-López et al. [19], who reported similar activity in the EVs of the amphizoic amoeba *A. culbertsoni*.

In this study, the interaction of EVs with epithelial cells was explored to determine whether EVs play a role in trophozoites facilitating crossing the epithelium prior to CNS invasion. Initially, it was determined that EVs decrease the transepithelial electrical resistance of monolayers, a result closely associated with amoeba pathogenesis, since Shibayama et al. [8] reported that a decrease in TER in the MDCK monolayer increases permeability, facilitating the invasion of trophozoites via the paracellular route. Based on our findings, we suggest that EVs could participate in this process.

Total *N. fowleri* extracts induce cell death via necrosis and apoptosis in microglia [56], consequently, determining whether EVs participate in this process is important. The results of confocal microscopy suggest that MDCK cells undergo necrosis 24 h after interaction with EVs. This result differs from that reported by Lertjuthaporn et al. [20], who did not detect macrophage necrosis during *N. fowleri* EVs interaction, probably because the EVs protein concentration (20 μg) was lower than that used in our work (40 μg). This finding corresponds with the observations of Goncalves et al. [18], who reported that the cytotoxic damage caused by *A. castellanii* EVs is dose dependent.

Following treatment with a serine protease inhibitor, a reduction in proteolytic activity was demonstrated in the zymograms; nonetheless, no reduction in the effects on haemolysis, transepithelial resistance, or cell death was observed. These findings suggest that other proteolytic factors may be involved in the observed cellular damage. Members of the calpain family, calcium-dependent proteases detected in EVs, could contribute to the damage observed in cell cultures. To further investigate the activity of these proteases, performing a zymogram with a casein substrate would be advisable [57]. In addition, phospholipases have been identified as cytolytic factors that contribute to cellular damage and are involved primarily in the breakdown of host proteins. This damage mechanism is common in bacteria [58, 59] and protozoa [60, 61] and facilitates host iron acquisition. *Naegleria fowleri* proteases have been shown to degrade human iron-binding proteins, including hololactoferrin, holotransferrin, and haemoglobin [62]. As an aerobic microorganism that causes haemorrhagic meningoencephalitis, *N. fowleri* is typically found near blood vessels via histopathological analyses [63]. This highlights the potential importance of the haemolytic activity observed in the EVs of amoebae in this study, which may be crucial for their pathogenicity.

*Naegleria fowleri* infection begins when trophozoites enter the nasal cavity, adhere to the mucosa, cross the nasal epithelium, and migrate to invade the CNS. One of the relevant steps in this process is the adhesion of the amoeba to the host tissue, which likely triggers a cascade of cellular signals leading to the expression of proteins and/or proteases. These molecules are pivotal in facilitating amoeba invasion into surrounding tissues [5]. We hypothesize that EV proteins may contribute to disrupting the blood-brain barrier, inducing cytotoxicity, and altering transepithelial resistance [10]. Additionally, the reduction in TER indicates that the effects of EVs partially contribute to the mechanical and/or enzymatic processes suggested in *A. castellanii*, where pathophysiological processes such as oedema and tissue architecture destabilization are determined [64].

Additionally, EVs may contribute to necrotic processes, facilitating amoebic invasion and proliferation in affected tissues. Our results suggest that the interaction of EVs with host cells contributes to the pathogenicity of these amoebae, which provides an understanding of the role of EVs in PAM. However, further research is needed to identify the specific factors responsible for the cytotoxic damage observed in these cells.

## Conclusions

*Naegleria fowleri* releases EVs of different sizes, which carry a wide range of proteins potentially involved in cell communication and virulence factor transfer, such as leishmanolysin. The internalization of these EVs by other *N. fowleri* trophozoites and MDCK epithelial cells reinforces these roles. Our results suggest that *N. fowleri* EVs play a role in pathogenic processes by inducing necrosis, increasing ionic paracellular permeability in MDCK cells, and causing haemolysis in human blood erythrocytes. However, further studies are needed to provide a more complete description of the role of the molecules contained in EVs in the pathogenesis of PAM and to elucidate their involvement in previously described processes, highlighting their mucolytic, adhesive, lipolytic, and phagocytic activities owing to their role in invasive processes to target tissues.

## Supplementary Information

Additional file 1: Fig. S1. Main protein groups contained in the extracellular vesicles (EVs) of *Naegleria fowleri*. The proteins most frequently found in EVs were metabolite interconversion enzymes and protein-modifying enzymes. The 'other proteins class' category included the following: RNA metabolism protein (1.8%), extracellular matrix protein (1.4%), calcium-binding protein (1.3%), defense/immunity protein (1.3%), transfer/carrier protein (0.8%), chromatin/chromatin-binding or regulatory protein (0.6%), DNA metabolism protein (0.3%), gene-specific transcriptional regulator (0.3%), cell adhesion protein (0.1%), intercellular signal molecule (0.1%) and transmembrane signal receptor (0.1%). The data were analysed using the PANTHER GO platform.

Additional file 2: Fig. S2. Gene Ontology analysis performed via PANTHER GO revealed terms associated with extracellular vesicles. (A) Molecular function terms. Among these terms, the following were enriched: catalytic and binding activities. The other molecular function terms were molecular adaptor activity (1.3%), translation regulator activity (1.2%), antioxidant activity (0.7%), electron transfer activity (0.4%), transcription regulator activity (0.3%), molecular transducer activity (0.3%), and cytoskeletal motor activity (0.1%). (B) Biological processes. In this graphic, proteins associated with cellular and metabolic processes stand out. The other biological process terms were developmental process (1.7%), multicellular organismal process (1%), homeostatic process (0.8%), locomotion (0.2%), pigmentation (0.2%), detoxification (0.1%), reproduction (0.1%), and reproductive process (0.1%). (C) Cellular components.

Additional file 3: Table S1. Proteins contained in *Naegleria fowleri* extracellular vesicles commonly reported in exosomes (ExoCarta: Exosome markers).

Additional file 4: Fig. S3. Haemolytic activity of extracellular vesicles (EVs). The absorbance results of the samples revealed a significant difference in the percentage of haemolytic cells in the presence of EVs compared with that of the negative control; however, there was no significant difference between the experimental groups of EVs with and without protease inhibitors. Negative control (-) erythrocytes in PBS. Positive control (+) erythrocytes with distilled water. The data were analysed using one-way ANOVA followed by Tukey’s post hoc test. The error bars represent the standard deviation of the mean. Asterisks indicate significant differences between the experimental groups and the control group (*P < 0.05). *n* = 3.

Additional file 5: Fig. S4. Effect of *Nagleria fowleri* extracellular vesicles (EVs) on the paracellular ionic permeability of MDCK cells. Transepithelial electrical resistance (TER) measurements were used to assess the ionic permeability of the paracellular pathway in the epithelium experimentally. Twenty-four hours after interaction, there was a significant decrease in the TER when MDCK cells interacted with *N. fowleri* EVs. These findings suggest that EVs increase paracellular ionic permeability. The data were analysed using one-way ANOVA followed by Tukey’s post hoc test. The error bars represent the standard deviation of the mean. Asterisks indicate significant differences between the experimental and control groups (*P < 0.05). *n* = 3.

## Data Availability

The mass spectrometry proteomics data have been deposited at the ProteomeXchange Consortium via the PRIDE [1] partner with the dataset identifier PXD059563.

## Abbreviations

CNS: Central nervous system
CHO cells: Chinese hamster ovary cells
DMEM: Dulbecco's modified Eagle medium
EDTA: Ethylenediaminetetraacetic acid
EVs: Extracellular vesicles
FBS: Fetal bovine serum
GP63: Glycoprotein 63
IA: Iodoacetamide
LC-MS: Liquid chromatography‒mass spectrometry
MDCK cells: Madin-Darby canine kidney cells
µg: Micrograms
MISEV: Minimal information for studies of extracellular vesicles
nm: Nanometers
NTA: Nanoparticle tracking analysis
PAM: Primary amebic meningoencephalitis
PAMPs: Pathogen-associated molecular patterns
PBS: Phosphate-buffered saline
PMSF: Phenylmethylsulfonyl fluoride
SD: Standard deviation
SDS-PAGE: Sodium dodecyl sulfate-polyacrylamide gel electrophoresis
TBST: Tris-buffered saline with Tween-20
TEM: Transmission electronic microscopy
TER: Transepithelial electrical resistance
ZO-1: Zonula occludens-1

## References

[1] Schuster FL, Visvesvara GS. Free-living amoebae as opportunistic and non-opportunistic pathogens of humans and animals. Int J Parasitol. 2004;34:1001–27. 15313128 10.1016/j.ijpara.2004.06.004

[2] Visvesvara GS. Infections with free-living amebae. In: Garcia HH, Tanowitz HB, Del Brutto OH, editors. Handbook of clinical neurology. Amsterdam: Elsevier B.V; 2013. p. 153–68. 10.1016/B978-0-444-53490-3.00010-8 23829906

[3] Zhang H, Cheng X. Various brain-eating amoebae: the protozoa, the pathogenesis, and the disease. Front Med. 2021. 10.1007/s11684-021-0865-2. 34825341 10.1007/s11684-021-0865-2

[4] Król-Turmińska K, Olender A. Human infections caused by free-living amoebae. Ann Agric Environ Med. 2017. 10.5604/12321966.1233568. 28664704 10.5604/12321966.1233568

[5] Güémez A, García E. Primary amoebic meningoencephalitis by *Naegleria fowleri*: pathogenesis and treatments. Biomolecules. 2021. 10.3390/biom11091320. 34572533 10.3390/biom11091320 PMC8469197

[6] Betanzos A, Bañuelos C, Orozco E. Host invasion by pathogenic amoebae: epithelial disruption by parasite proteins. Genes. 2019. 10.3390/genes10080618. 31416298 10.3390/genes10080618 PMC6723116

[7] Han KL, Lee HJ, Myeong HS, Shin HJ, Im K-I, Park SJ. The involvement of an integrin-like protein and protein kinase C in amoebic adhesion to fibronectin and amoebic cytotoxicity. Parasitol Res. 2004. 10.1007/s00436-004-1158-9. 15338291 10.1007/s00436-004-1158-9

[8] Shibayama M, Martínez-Castillo M, Silva-Olivares A, Galindo-Gómez S, Navarro-García F, Escobar-Herrera J, et al. Disruption of MDCK cell tight junctions by the free-living amoeba *Naegleria fowleri*. Microbiology. 2013. 10.1099/mic.0.063255-0. 23258265 10.1099/mic.0.063255-0

[9] Cervantes-Sandoval I, de Serrano-Luna JJ, García-Latorre E, Tsutsumi V, Shibayama M. Mucins in the host defence against *Naegleria fowleri* and mucinolytic activity as a possible means of evasion. Microbiology. 2008. 10.1099/mic.0.2008/019380-0. 19047756 10.1099/mic.0.2008/019380-0

[10] Coronado-Velázquez D, Betanzos A, Serrano-Luna J, Shibayama M. An In vitro model of the Blood-Brain Barrier: *Naegleria fowleri* affects the tight junction proteins and activates the microvascular endothelial cells. J Eukaryot Microbiol. 2018. 10.1111/jeu.12522. 29655298 10.1111/jeu.12522

[11] Young JDE, Lowrey DM. Biochemical and functional characterization of a membrane-associated pore-forming protein from the pathogenic ameboflagellate *Naegleria fowleri*. J Biol Chem. 1989;264:1077–83. 2463245

[12] Herbst R, Ott C, Jacobs T, Marti T, Marciano-Cabral F, Leippe M. Pore-forming polypeptides of the pathogenic protozoon *Naegleria fowleri*. J Biol Chem. 2002. 10.1074/jbc.M201475200. 11948186 10.1074/jbc.M201475200

[13] Chávez-Munguía B, Salazar Villatoro L, Omaña-Molina M, Rodríguez-Monroy MA, Segovia-Gamboa N, Martínez-Palomo A. *Naegleria fowleri*: contact-dependent secretion of electrondense granules (EDG). Exp Parasitol. 2014. 10.1016/j.exppara.2014.03.027. 24721258 10.1016/j.exppara.2014.03.027

[14] Théry C, Witwer KW, Aikawa E, Alcaraz MJ, Anderson JD, Andriantsitohaina R, et al. Minimal information for studies of extracellular vesicles 2018 (MISEV2018): a position statement of the International Society for Extracellular Vesicles and update of the MISEV2014 guidelines. J Extracell Vesicles. 2018. 10.1080/20013078.2018.1535750. 30637094 10.1080/20013078.2018.1535750 PMC6322352

[15] Nievas YR, Lizarraga A, Salas N, Cóceres VM, de Miguel N. Extracellular vesicles released by anaerobic protozoan parasites: current situation. Cell Microbiol. 2020. 10.1111/cmi.13257. 32858768 10.1111/cmi.13257

[16] Welsh JA, Goberdhan DCI, O’Driscoll L, Buzas EI, Blenkiron C, Bussolati B, et al. Minimal information for studies of extracellular vesicles (MISEV2023): from basic to advanced approaches. J Extracell Vesicles. 2024. 10.1002/jev2.12404. 38326288 10.1002/jev2.12404 PMC10850029

[17] Twu O, de Miguel N, Lustig G, Stevens GC, Vashisht AA, Wohlschlegel JA, et al. *Trichomonas vaginalis* exosomes deliver cargo to host cells and mediate host: parasite interactions. PLoS Pathog. 2013. 10.1371/journal.ppat.1003482. 23853596 10.1371/journal.ppat.1003482 PMC3708881

[18] de Souza GD, da Silva FM, Liedke SC, Gomes KX, de Oliveira GA, Leão PEL, et al. Extracellular vesicles and vesicle-free secretome of the protozoa *Acanthamoeba castellanii* under homeostasis and nutritional stress and their damaging potential to host cells. Virulence. 2018. 10.1080/21505594.2018.1451184. 10.1080/21505594.2018.1451184 PMC5955443 29560793

[19] Sierra-López F, Castelan-Ramírez I, Hernández-Martínez D, Salazar-Villatoro L, Segura-Cobos D, Flores-Maldonado C, et al. Extracellular vesicles secreted by *Acanthamoeba culbertsoni* have COX and proteolytic activity and induce hemolysis. Microorganisms. 2023. 10.3390/microorganisms11112762. 38004773 10.3390/microorganisms11112762 PMC10673465

[20] Lertjuthaporn S, Somkird J, Lekmanee K, Atipimonpat A, Sukapirom K, Sawasdipokin H, et al. Extracellular vesicles from *Naegleria fowleri* induce IL-8 response in THP-1 macrophage. Pathogens. 2022. 10.3390/pathogens11060632. 35745486 10.3390/pathogens11060632 PMC9231210

[21] Retana Moreira L, Steller Espinoza MF, Chacón Camacho N, Cornet-Gomez A, Sáenz-Arce G, Osuna A, et al. Characterization of extracellular vesicles secreted by a clinical isolate of *Naegleria fowleri* and identification of immunogenic components within their protein cargo. Biology. 2022. 10.3390/biology11070983. 36101365 10.3390/biology11070983 PMC9312180

[22] Lê HG, Kang JM, Võ TC, Yoo WG, Na BK. *Naegleria fowleri* extracellular vesicles induce proinflammatory immune responses in BV-2 Microglial Cells. Int J Mol Sci. 2023. 10.3390/ijms241713623. 37686429 10.3390/ijms241713623 PMC10487526

[23] Russell AC, Bush P, Grigorean G, Kyle DE. Characterization of the extracellular vesicles, ultrastructural morphology, and intercellular interactions of multiple clinical isolates of the brain-eating amoeba, *Naegleria fowleri*. Front Microbiol. 2023. 10.3389/fmicb.2023.1264348. 37808283 10.3389/fmicb.2023.1264348 PMC10558758

[24] Retana Moreira L, Cornet-Gomez A, Sepulveda MR, Molina-Castro S, Alvarado-Ocampo J, Chaves Monge F, et al. Providing an in vitro depiction of microglial cells challenged with immunostimulatory extracellular vesicles of *Naegleria fowleri*. Front Microbiol. 2024. 10.3389/fmicb.2024.1346021. 38374922 10.3389/fmicb.2024.1346021 PMC10876093

[25] Shevchenko A, Tomas H, Havliš J, Olsen JV, Mann M. In-gel digestion for mass spectrometric characterization of proteins and proteomes. Nat Protoc. 2007. 10.1038/nprot.2006.468. 10.1038/nprot.2006.468 17406544

[26] Barrera-Rojas J, Gurubel-Tun KJ, Ríos-Castro E, López-Méndez MC, Sulbarán-Rangel B. An initial proteomic analysis of biogas-related metabolism of *Euryarchaeota* consortia in sediments from the Santiago River, México. Microorganisms. 2023. 10.3390/microorganisms11071640. 37512813 10.3390/microorganisms11071640 PMC10384328

[27] Rios-Castro E, Souza GHMF, Delgadillo-Alvarez DM, Ramirez-Reyes L, Torres-Huerta AL, Velasco-Suarez A, et al. Quantitative proteomic analysis of MARC-145 cells infected with a Mexican Porcine Reproductive and Respiratory Syndrome Virus strain using a Label-Free Based DIA approach. J Am Soc Mass Spectrom. 2020. 10.1021/jasms.0c00134. 32379441 10.1021/jasms.0c00134

[28] Li GZ, Vissers JPC, Silva JC, Golick D, Gorenstein MV, Geromanos SJ. Database searching and accounting of multiplexed precursor and product ion spectra from the data independent analysis of simple and complex peptide mixtures. Proteomics. 2009. 10.1002/pmic.200800564. 19294629 10.1002/pmic.200800564

[29] Käll L, Storey JD, MacCoss MJ, Noble WS. Assigning significance to peptides identified by tandem mass spectrometry using decoy databases. J Proteome Res. 2008. 10.1021/pr700600n. 18067246 10.1021/pr700600n

[30] Pierson T, Matrakas D, Taylor YU, Manyam G, Morozov VN, Zhou W, et al. Proteomic characterization and functional analysis of outer membrane vesicles of *Francisella novicida* suggests possible role in virulence and use as a vaccine. J Proteome Res. 2011. 10.1021/pr1009756. 21138299 10.1021/pr1009756

[31] Sæbø I, Bjørås M, Franzyk H, Helgesen E, Booth J. Optimization of the hemolysis assay for the assessment of cytotoxicity. Int J Mol Sci. 2023. 10.3390/ijms24032914. 36769243 10.3390/ijms24032914 PMC9917735

[32] Cereijido M, Robbins E, Dolan W, Rotunno C, Sabatini D. Polarized monolayers formed by epithelial cells on a permeable and translucent support. J Cell Biol. 1978. 10.1083/jcb.77.3.853. 567227 10.1083/jcb.77.3.853 PMC2110138

[33] Cereijido M, Meza I, Martinez-Palomo A. Occluding junctions in cultured epithelial monolayers. Am J Physiol. 1981. 10.1152/ajpcell.1981.240.3.C96. 7212057 10.1152/ajpcell.1981.240.3.C96

[34] Cereijido M, González-Mariscal L, Contreras RG, Gallardo JM, Garcia-Villegas R, Valdés J. The making of a tight junction. J Cell Sci Suppl. 1993. 10.1242/jcs.1993.supplement_17.18. 8144687 10.1242/jcs.1993.supplement_17.18

[35] Cereijido M, Ehrenfeld J, Meza I, Martínez-Palomo A. Structural and functional membrane polarity in cultured monolayers of MDCK cells. J Membr Biol. 1980. 10.1007/BF01869120. 6245216 10.1007/BF01869120

[36] Cereijido M, González-Mariscal L, Borboa L. Occluding junctions and paracellular pathways studied in monolayers of MDCK cells. J Exp Biol. 1983. 10.1242/jeb.106.1.205. 6686247 10.1242/jeb.106.1.205

[37] Araki T, Hayashi M, Watanabe N, Kanuka H, Yoshino J, Miura M, et al. Down-Regulation of Mcl-1 by inhibition of the PI3-K/Akt pathway is required for cell shrinkage-dependent cell death. Biochem Biophys Res Commun. 2002. 10.1006/bbrc.2002.6329. 11812001 10.1006/bbrc.2002.6329

[38] Van Buren T, Arwatz G, Smits AJ. A simple method to monitor hemolysis in real time. Sci Rep. 2020. 10.1038/s41598-020-62041-8. 32198369 10.1038/s41598-020-62041-8 PMC7083869

[39] Kalra H, Drummen GPC, Mathivanan S. Focus on extracellular vesicles: introducing the next small big thing. Int J Mol Sci. 2016. 10.3390/ijms17020170. 26861301 10.3390/ijms17020170 PMC4783904

[40] Kelsall BL, Ravdin JI. Immunization of rats with the 260-kilodalton *Entamoeba histolytica* galactose-inhibitable lectin elicits an intestinal secretory immunoglobulin a response that has in vitro adherence-inhibitory activity. Infect Immun. 1995. 10.1128/iai.63.2.686-689.1995. 7822040 10.1128/iai.63.2.686-689.1995 PMC173050

[41] Dixson AC, Dawson TR, Di Vizio D, Weaver AM. Context-specific regulation of extracellular vesicle biogenesis and cargo selection. Nat Rev Mol Cell Biol. 2023. 10.1038/s41580-023-00576-0. 36765164 10.1038/s41580-023-00576-0 PMC10330318

[42] Martínez AJ, Visvesvara GS. Pathogenic and opportunistic free-living amebas: *Naegleria fowleri*, *Acanthamoeba* spp. and *Balamuthia mandrillaris*. In: Gillespie S, Pearson RD, editors. Principles and practice of clinical parasitology. Hoboken: Wiley; 2001. p. 269–85.

[43] Mercado-Camargo J, Cervantes-Ceballos L, Vivas-Reyes R, Vivas-Reyes R, Vivas-Reyes R, Pedretti A, et al. Homology modeling of Leishmanolysin (gp63) from *Leishmania panamensis* and molecular docking of flavonoids. ACS Omega. 2020. 10.1021/acsomega.0c01584. 32596611 10.1021/acsomega.0c01584 PMC7315592

[44] Oliveira SSC, Correia CA, Santos VS, da Cunha EFF, de Castro AA, Ramalho TC, et al. Silver(I) and Copper(II) 1,10-Phenanthroline-5,6-dione complexes as promising antivirulence strategy against *Leishmania*: focus on Gp63 (Leishmanolysin). Trop Med Infect Dis. 2023. 10.3390/tropicalmed8070348. 37505644 10.3390/tropicalmed8070348 PMC10384183

[45] Hernández-Ramírez VI, Estrada-Figueroa LA, Medina Y, Lizarazo-Taborda MR, Toledo-Leyva A, Osorio-Trujillo C, et al. A monoclonal antibody against a *Leishmania mexicana* COX-like enzymatic activity also recognizes similar proteins in different protozoa of clinical importance. Parasitol Res. 2023. 10.1007/s00436-022-07746-7. 36562799 10.1007/s00436-022-07746-7

[46] de Silva Lira Filho A, Fajardo EF, Chang KP, Clément P, Olivier M. *Leishmania* exosomes/extracellular vesicles containing GP63 are essential for enhance cutaneous Leishmaniasis development upon co-inoculation of *Leishmania amazonensis* and its exosomes. Front Cell Infect Microbiol. 2022. 10.3389/fcimb.2021.709258. 35186777 10.3389/fcimb.2021.709258 PMC8851419

[47] Wang X, Chen J, Zheng J. The roles of COX-2 in protozoan infection. Front Immunol. 2023. 10.3389/fimmu.2023.955616. 10.3389/fimmu.2023.955616 PMC9978824 36875123

[48] Aldape K, Huizinga H, Bouvier J, McKerrow J. *Naegleria fowleri*: characterization of a secreted histolytic cysteine protease. Exp Parasitol. 1994. 10.1006/expr.1994.1023. 8119377 10.1006/expr.1994.1023

[49] Fitzner D, Schnaars M, Van Rossum D, Krishnamoorthy G, Dibaj P, Bakhti M, et al. Selective transfer of exosomes from oligodendrocytes to microglia by macropinocytosis. J Cell Sci. 2011. 10.1242/jcs.074088. 21242314 10.1242/jcs.074088

[50] Douanne N, Dong G, Amin A, Bernardo L, Blanchette M, Langlais D, et al. *Leishmania* parasites exchange drug-resistance genes through extracellular vesicles. Cell Rep. 2022. 10.1016/j.celrep.2022.111121. 35858561 10.1016/j.celrep.2022.111121

[51] Mulcahy LA, Pink RC, Carter DRF. Routes and mechanisms of extracellular vesicle uptake. J Extracell Vesicles. 2014. 10.3402/jev.v3.24641. 25143819 10.3402/jev.v3.24641 PMC4122821

[52] Martínez-Castillo M, Santos-Argumedo L, Galván-Moroyoqui JM, Serrano-Luna J, Shibayama M. Toll-like receptors participate in *Naegleria fowleri* recognition. Parasitol Res. 2018. 10.1007/s00436-017-5666-9. 29128927 10.1007/s00436-017-5666-9

[53] Maldonado-Bernal C, Kirschning CJ, Rosenstein Y, Rocha LM, Rios-Sarabia N, Espinosa-Cantellano M, et al. The innate immune response to *Entamoeba histolytica* lipopeptidophosphoglycan is mediated by toll-like receptors 2 and 4. Parasite Immunol. 2005. 10.1111/j.1365-3024.2005.00754.x. 15910421 10.1111/j.1365-3024.2005.00754.x

[54] Chadee K, Petri WA, Innes DJ, Ravdin JI. Rat and human colonic mucins bind to and inhibit adherence lectin of *Entamoeba histolytica*. J Clin Invest. 1987. 10.1172/JCI113199. 2890655 10.1172/JCI113199 PMC442377

[55] Underhill DM, Gantner B. Integration of Toll-like receptor and phagocytic signaling for tailored immunity. Microbes Infect. 2004. 10.1016/j.micinf.2004.08.016. 15596122 10.1016/j.micinf.2004.08.016

[56] Kim JH, Kim D, Shin HJ. Contact-Independent cell Death of human microglial cells due to pathogenic *Naegleria fowleri* trophozoites. Korean J Parasitol. 2008. 10.3347/kjp.2008.46.4.217. 19127326 10.3347/kjp.2008.46.4.217 PMC2612605

[57] Wang KKW. Calpain zymography: general methodology and protocol. In: Wilkesman J, Kurz L, editors. Methods in molecular biology. Totowa: Humana Press Inc.; 2017. p. 279–85. 10.1007/978-1-4939-7111-4_26 28608220

[58] Skaar EP. The battle for Iron between bacterial pathogens and their vertebrate hosts. PLoS Pathog. 2010. 10.1371/journal.ppat.1000949. 20711357 10.1371/journal.ppat.1000949 PMC2920840

[59] Morgenthau A, Pogoutse A, Adamiak P, Moraes TF, Schryvers AB. Bacterial receptors for host transferrin and lactoferrin: molecular mechanisms and role in host–microbe interactions. Future Microbiol. 2013. 10.2217/fmb.13.125. 24266357 10.2217/fmb.13.125

[60] León-Sicairos N, Reyes-López M, Canizalez-Román A, Bermúdez-Cruz RM, Serrano-Luna J, Arroyo R, et al. Human hololactoferrin: endocytosis and use as an iron source by the parasite *Entamoeba histolytica*. Microbiology. 2005. 10.1099/mic.0.28121-0. 16339932 10.1099/mic.0.28121-0

[61] Ortíz-Estrada G, Luna-Castro S, Piña-Vázquez C, Samaniego-Barrón L, León-Sicairos N, Serrano-Luna J, et al. Iron-saturated lactoferrin and pathogenic protozoa: could this protein be an iron source for their parasitic style of life? Future Microbiol. 2012. 10.2217/fmb.11.140. 22191452 10.2217/fmb.11.140

[62] Martínez-Castillo M, Ramírez-Rico G, Serrano-Luna J, Shibayama M. Iron-binding protein degradation by cysteine proteases of *Naegleria fowleri*. Biomed Res Int. 2015. 10.1155/2015/416712. 26090408 10.1155/2015/416712 PMC4450812

[63] Visvesvara GS, Moura H, Schuster FL. Pathogenic and opportunistic free-living amoebae: *Acanthamoeba* spp., *Balamuthia mandrillaris*, *Naegleria fowleri*, and *Sappinia diploidea*. FEMS Immunol Med Microbiol. 2007. 10.1111/j.1574-695X.2007.00232.x. 17428307 10.1111/j.1574-695X.2007.00232.x

[64] Omaña-Molina M, González-Robles A, Iliana Salazar-Villatoro L, Lorenzo-Morales J, Cristóbal-Ramos AR, Hernández-Ramírez VI, et al. Reevaluating the role of *Acanthamoeba* proteases in tissue invasion: observation of cytopathogenic mechanisms on MDCK Cell monolayers and hamster corneal cells. Biomed Res Int. 2013. 10.1155/2013/461329. 23484119 10.1155/2013/461329 PMC3581277

