# Supplementary figures and images for: Advances in the study of extracellular vesicles of Naegleria fowleri and their role in contact-independent pathogenic mechanisms
Source: Parasit Vectors. 2025 May 1;18:164. doi: 10.1186/s13071-025-06786-z (PMC12046931; doi:10.1186/s13071-025-06786-z)

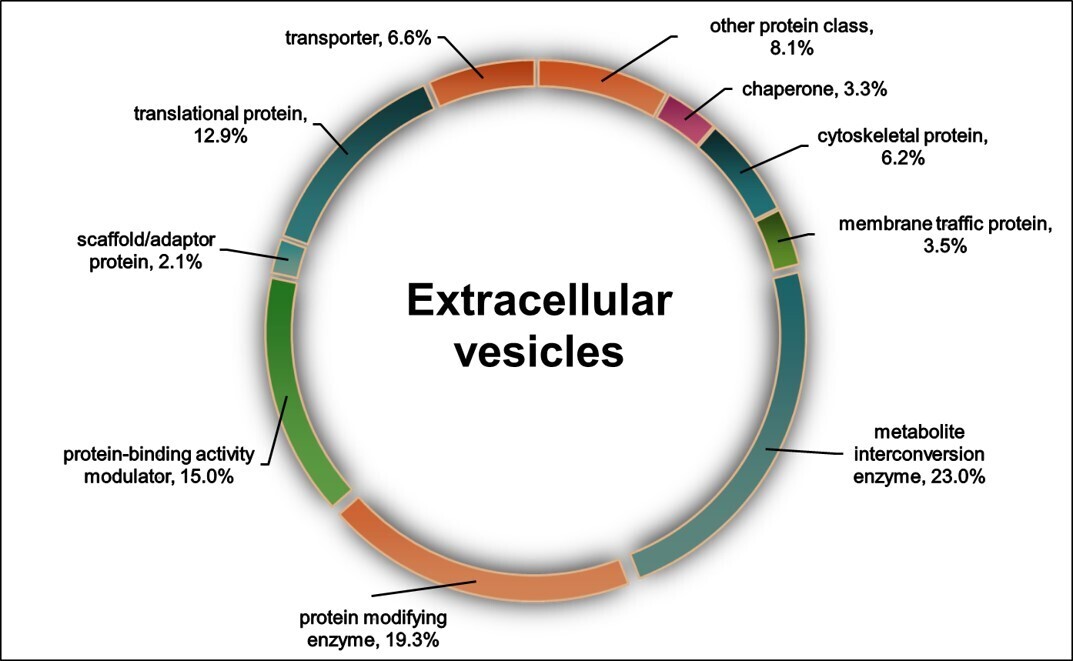

Supplement: Supplementary file 1 — Additional file 1: Fig. S1. Main protein groups contained in the extracellular vesicles (EVs) of Naegleria fowleri. The proteins most frequently found in EVs were metabolite interconversion enzymes and protein-modifying enzymes. The 'other proteins class' category included the following: RNA metabolism protein (1.8%), extracellular matrix protein (1.4%), calcium-binding protein (1.3%), defense/immunity protein (1.3%), transfer/carrier protein (0.8%), chromatin/chromatin-binding or regulatory protein (0.6%), DNA metabolism protein (0.3%), gene-specific transcriptional regulator (0.3%), cell adhesion protein (0.1%), intercellular signal molecule (0.1%) and transmembrane signal receptor (0.1%). The data were analysed using the PANTHER GO platform. [file 13071_2025_6786_MOESM1_ESM.jpeg]

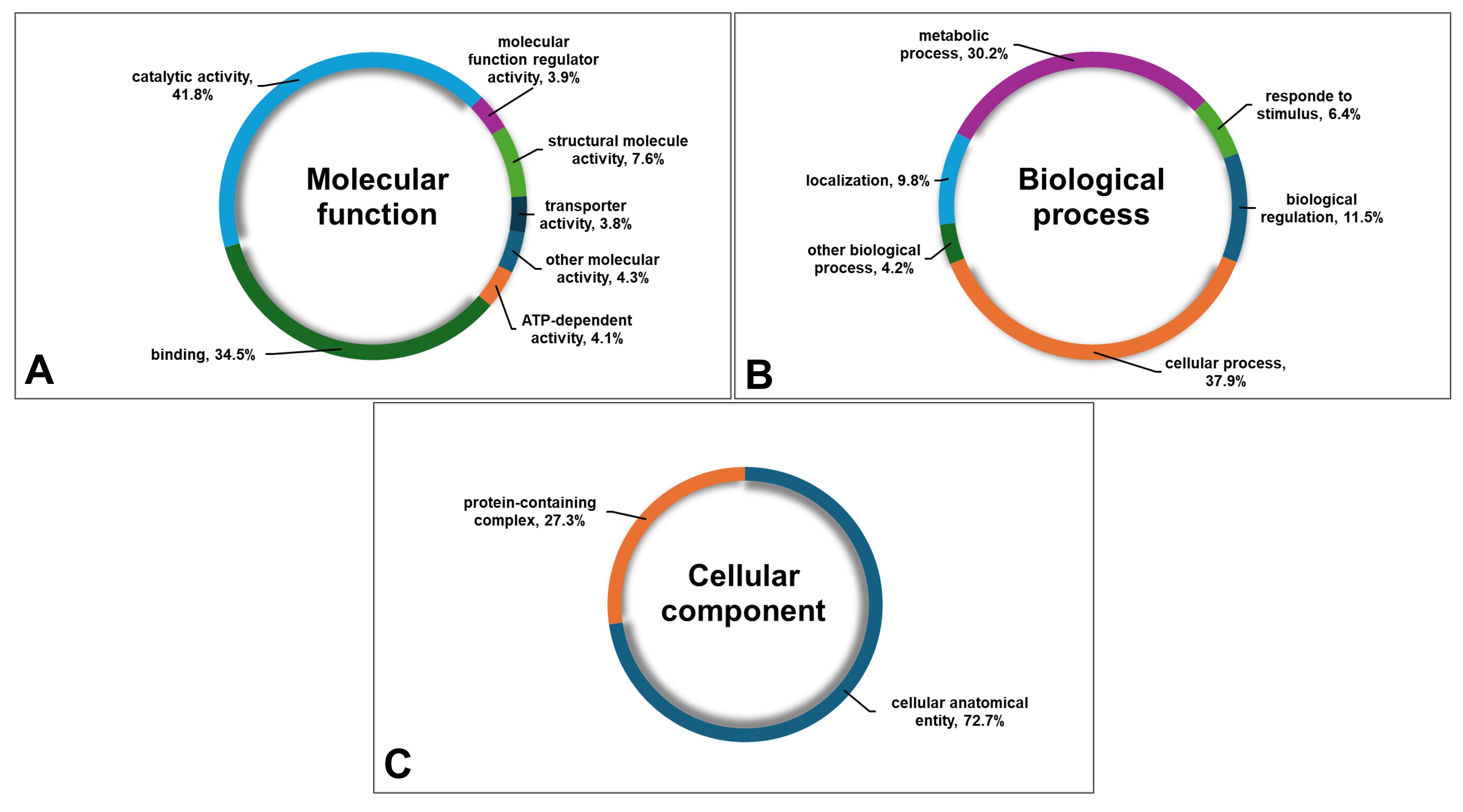

Supplement: Supplementary file 2 — Additional file 2: Fig. S2. Gene Ontology analysis performed via PANTHER GO revealed terms associated with extracellular vesicles. (A) Molecular function terms. Among these terms, the following were enriched: catalytic and binding activities. The other molecular function terms were molecular adaptor activity (1.3%), translation regulator activity (1.2%), antioxidant activity (0.7%), electron transfer activity (0.4%), transcription regulator activity (0.3%), molecular transducer activity (0.3%), and cytoskeletal motor activity (0.1%). (B) Biological processes. In this graphic, proteins associated with cellular and metabolic processes stand out. The other biological process terms were developmental process (1.7%), multicellular organismal process (1%), homeostatic process (0.8%), locomotion (0.2%), pigmentation (0.2%), detoxification (0.1%), reproduction (0.1%), and reproductive process (0.1%). (C) Cellular components. [file 13071_2025_6786_MOESM2_ESM.jpeg]

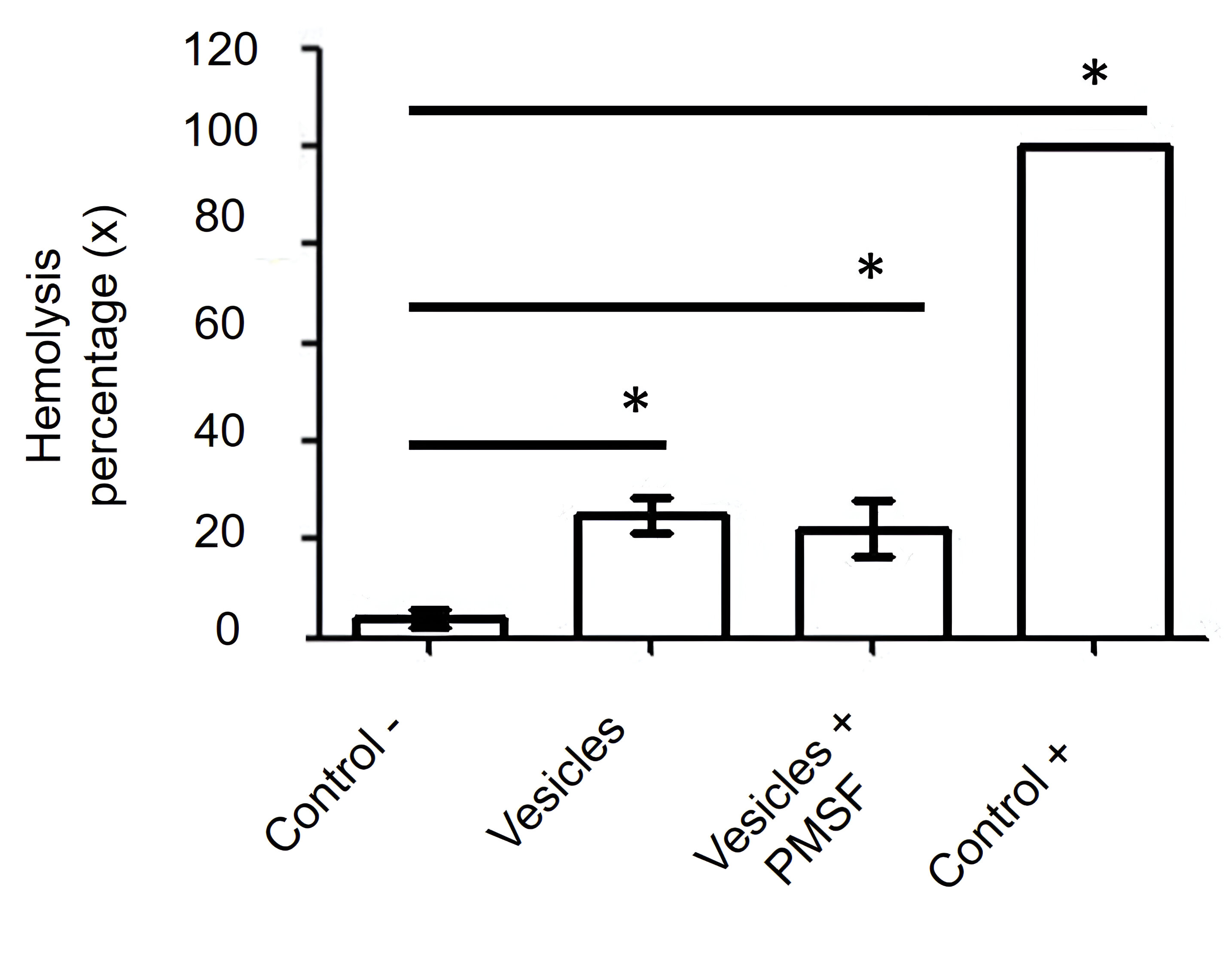

Supplement: Supplementary file 4 — Additional file 4: Fig. S3. Haemolytic activity of extracellular vesicles (EVs). The absorbance results of the samples revealed a significant difference in the percentage of haemolytic cells in the presence of EVs compared with that of the negative control; however, there was no significant difference between the experimental groups of EVs with and without protease inhibitors. Negative control (-) erythrocytes in PBS. Positive control (+) erythrocytes with distilled water. The data were analysed using one-way ANOVA followed by Tukey’s post hoc test. The error bars represent the standard deviation of the mean. Asterisks indicate significant differences between the experimental groups and the control group (*P < 0.05). n = 3. [file 13071_2025_6786_MOESM4_ESM.jpeg]

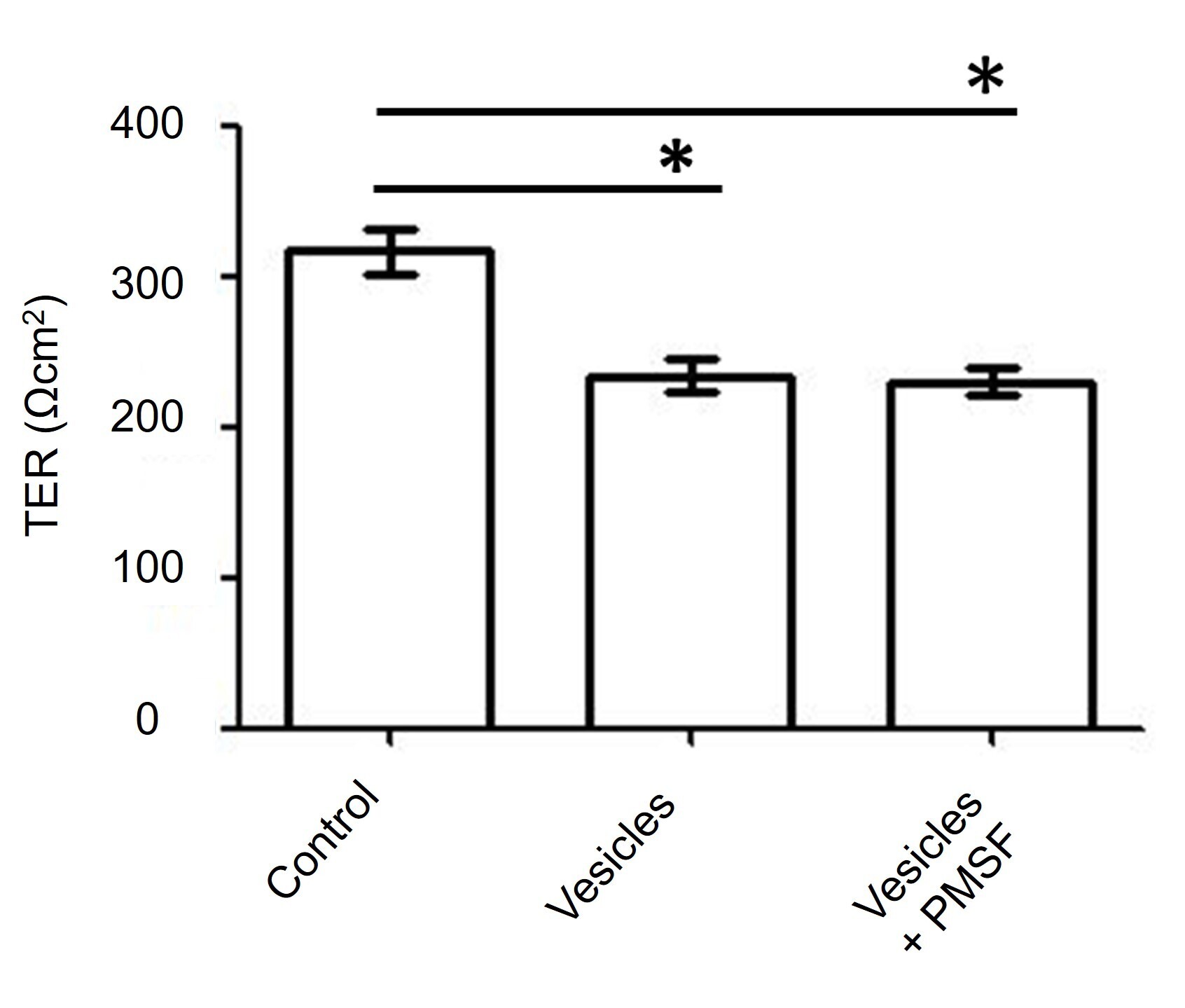

Supplement: Supplementary file 5 — Additional file 5: Fig. S4. Effect of Nagleria fowleri extracellular vesicles (EVs) on the paracellular ionic permeability of MDCK cells. Transepithelial electrical resistance (TER) measurements were used to assess the ionic permeability of the paracellular pathway in the epithelium experimentally. Twenty-four hours after interaction, there was a significant decrease in the TER when MDCK cells interacted with N. fowleri EVs. These findings suggest that EVs increase paracellular ionic permeability. The data were analysed using one-way ANOVA followed by Tukey’s post hoc test. The error bars represent the standard deviation of the mean. Asterisks indicate significant differences between the experimental and control groups (*P < 0.05). n = 3. [file 13071_2025_6786_MOESM5_ESM.jpeg]
