# Supplementary material for: Advances in the study of extracellular vesicles of Naegleria fowleri and their role in contact-independent pathogenic mechanisms
Source: Parasit Vectors. 2025 May 1;18:164. doi: 10.1186/s13071-025-06786-z (PMC12046931; doi:10.1186/s13071-025-06786-z)
Supplement: Supplementary file 3 — Additional file 3: Table S1. Proteins contained in Naegleria fowleri extracellular vesicles commonly reported in exosomes (ExoCarta: Exosome markers). [file 13071_2025_6786_MOESM3_ESM.docx]

| **Protein** | **Accession** | **Position in Exocharter** |
| --- | --- | --- |
| 70 kDa heat shock protein (HSP 70) | A0A6A5BL70 | 2 |
| Glyceraldehyde-3-phosphate dehydrogenase (GAPDH) | A0A4V8H039 | 4 |
| Phosphoglycerate kinase (PK) | A0A6A5BXT8 | 16 |
| Fructose-bisphosphate aldolase | A0A6A5BF81 | 18 |
| Triosephosphate isomerase | A0A6A5BWU3 | 27 |
| Profilin | A0A6A5BHQ3 | 32 |
